# Carbothioamide functionalized paroxetine analogues as antiparasitic agents targeting the hypoxanthine-guanine-xanthine phosphoribosyltransferase enzyme: Synthesis, characterization, biochemical, biophysical, and *in silico* studies

**DOI:** 10.1371/journal.pone.0359519

**Published:** 2026-10-22

**Authors:** Laleen Hammal, Mukhtiyar Ali, Naveera Zafar, Saima Hassan Mari, Humaira Zafar, Atia-tul- Wahab, Hina Siddiqui, Sumaira Javaid, Muhammad Iqbal Choudhary

**Affiliations:** 1 Dr. Panjwani Center for Molecular Medicine and Drug Research, International Center of Chemical and Biological Sciences, University of Karachi, Karachi, Pakistan; 2 H. E. J. Research Institute of Chemistry, International Center for Chemical and Biological Sciences, University of Karachi, Karachi, Pakistan; 3 Institute of Molecular Biology and Biochemistry, The University of Lahore, Lahore Pakistan; 4 Institute of Drug Discovery Technology, Ningbo University, Ningbo, Zhejiang, China; Universiti Teknologi Malaysia, MALAYSIA

## Abstract

Hypoxanthine guanine xanthine phosphoribosyl transferase (HGXPRT) is one of the central enzymes of purine salvage pathway of protozoan parasites, including *Plasmodium*. These parasites are purine auxotrophs, and rely on purine salvage pathway for nucleic acid synthesis. Inhibiting the enzymatic activity can effectively reduce the parasite- induced infections, including malaria. In this context, a library of new carbothioamide derivatives **1–17** of paroxetine was synthesized *via* single-step chemical transformation strategy with a series of substituted phenyl isothiocyanates. All compounds were purified, and their structures were deduced by using spectroscopic techniques, such as mass, FT-IR, UV, and ^1^H-, and ^13^C-NMR. These compounds were evaluated for their HGXPRT inhibitory activity *via in vitr*o UV-based spectrophotometric assay. All derivatives **1**–**17** showed varying potential to inhibit the enzyme activity with IC_50_ values between 19.0 to 76.9 µM, with highest activity showed by compound **13** (IC_50_ = 19.0 ± 2.09 µM) within the series, but remained less potent than the standard (9-deazaguanine, IC_50_ = 12.00 ± 1.0 µM). Biophysical, molecular docking, and molecular simulation studies were carried out further to analyse the enzyme-inhibitor interaction, and stability. These compounds were predicted to occupy the putative allosteric site of enzyme. Furthermore, cytotoxic profile of these compounds were also evaluated *via* ADMET analysis and MTT assay, and found to be largely non-cytotoxic. The current study has identified potential hits against HGXPRT enzyme that could further be investigated as anti-parasitic drugs.

## Introduction

Hypoxanthine-guanine-xanthine phosphoribosyltransferase (HGXPRT; EC 2.4.2.8) is one of central enzymes of purine salvage pathway. It plays a key role in enabling the parasite to acquire nucleotides, particularly purine, for the DNA and RNA synthesis, from the host. Protozoan parasites, including *Plasmodium* being purine auxotroph cannot synthesize the nucleotides *de novo*, therefore enzyme HGXPRT help the parasite to replicate, proliferate, and survive within human host [1,2]. HGXPRT enzyme is responsible to incorporate the purine bases, such as hypoxanthine, guanine, and xanthine, into the nucleotide pool of the parasite, particularly hypoxanthine is a key precursor for the biosynthesis of nucleotides [3]. Hypoxanthine and inosine are formed in the erythrocyte of host, and are transferred to the parasites through the purine base, and nucleoside transporters, present in the parasite membrane. HGXPRT is located in the cytoplasm of the *Plasmodium* species. It is a magnesium-dependent *bi*-substrate enzyme that catalyses the transfer of phosphoribosyl group from α-D-5-phopshoribosyl-1-pyrophosphate (PRPP) to purine bases, and generate their respective nucleotides [2]. It is reported that the binding of substrates to the enzyme is highly ordered, where PRPP is being the first substrate to bind, followed by purine binding.

HGXPRT enzyme is vital to the survival of protozoan parasites of genus *Plasmodium*, which cause malaria, a serious vector-borne infectious diseases [1]. Malaria is a major global health concern, responsible of over 250 million clinical cases with 610,000 deaths in 2024 as per WHO report [4]*.* Climate change, inadequate healthcare infrastructure, and emerging resistance against antimalarial drugs are the key factors that contribute in malaria’s persistence and spread [4]. This scenario necessitates the identification of new drugs that can combat malaria effectively. Several classes of compounds have been reported as HGXPRT inhibitors, such as pyrrolidine nucleoside mono-, and *bis*-phosphonates, immucillin 5’-monophosphate derivatives, aza-acyclic nucleoside phosphonates, acyclic immucillin phosphonates, quinoline-based hybrid compounds, 4-phthalimidocarboxanilides and prolinol connected to purine base, etc [5,6]. However, many of them are associated with severe cytotoxicity [5–7]. Therefore, development of new inhibitors with least cytotoxicity is imperative in disrupting purine recycling in pathogenic parasite, and thus diminishing their ability to replicate, and survive.

For this purpose, we have synthesized a series of paroxetine-carbothioamide derivatives *via* single-step chemical transformation. Paroxetine is a US-FDA-approved anti-depressant drug, and its structure consists of piperidine ring, a benzodioxol, and a fluorophenyl groups. It was selected as the base scaffold due to its well-established pharmacokinetic properties. Presence of a reactive secondary amine, enable facile carbothioamide (thiourea) formation, and its rigid structure supports the favourable interactions with biological targets. Additionally, incorporation of the carbothioamide moiety is known for its antiparasitic activity [8–11], and the potential for scaffold repurposing make paroxetine a suitable candidate for designing new antimalarial agents. It can also act as a bioisostere for carboxamides, and improve enzyme binding. Thus combining paroxetine and carbothioamide together can create hybrid inhibitors with the potential to bind effectively to the purine binding site of HGXPRT enzyme, and inhibits its biological function.

During the current study, the HGXPRT enzyme was produced *via* recombinant technology, following the method reported earlier [12,13]. This was followed by the systematic screening of paroxetine-carbothioamide derivatives. Compounds with *in vitro* anti-HGXPRT activity were further studied *via* biophysical and *in silico* techniques to predict enzyme-inhibitor interactions, binding affinity, and the stability of the enzyme-inhibitor complex. These compounds were also subjected to cytotoxic analysis against human fibroblast cell line to assess their safety profile.

## Materials and methods

### Chemicals for the synthesis of compounds

Paroxetine **(a)** was purified from the tablets “Seroxate” (GlaxoSmithKline, Pakistan). 3-Trifluoromethylphenyl, 3-bromophenyl, 4-fluorophenyl, and 3-nitrophenyl isothiocyanate were obtained from Sigma Aldrich (Poland), while 3-fluorophenyl, 2,5-dichlorophenyl, and 4-methoxyphenyl isothiocyanates were purchased from Sigma Aldrich (USA). 4-Chlorophenyl, 3-chlorophenyl, 2,4-difluorophenyl, and 3,4-dichlorophenyl isothiocyanate from Alfa Aesar (USA), while 2-fluorophenyl isothiocyanate from Sigma Aldrich Chemie (Germany). 4-Trifluoromethoxy phenyl- and 4-cyanophenyl-isothiocyanates were obtained from Oakwood Chemical Products (USA). The other isothiocyanates, such as phenyl, as well as 3,4-difluorophenyl isothiocyanate, were purchased from TCI (Japan). Silica-based preparative HPLC column (D-60, 20 mm × 250 mm, 5 µm; YMC Co., Ltd., Japan), and thin layer chromatography silica gel plates (ALUGRAM, SIL G/UV254) were used for separating and identifying components of a mixture. For HPLC, detection, ultraviolet (UV) and refractive index (RI) systems were used while TLC chromatogram was examined by using UV light at 254, and 365 nm. ESI-MS and FAB-MS mass spectra were recorded by using a JEOLJ MS-600H mass spectrometer (Japan). For NMR studies samples were prepared at a concentration of approx. 3 mg/ mL in DMSO-_*d*_*_6_*. Bruker Neo spectrometers (Switzerland) were used to record ^1^H-NMR at 400 and 500 MHz, and ^13^C-NMR spectra at 100, and 125 MHz. All experiments were performed at 298 K. No solvent suppression was applied. The melting points were measured by using the Buchi M-560 apparatus (Japan). FT-IR spectra were recorded on an FTIR 8900 (Shimadzu, Japan) using a KBr disc.

### Chemicals for the biological activity

*E. coli* BL21(DE3) pLysS cells (Cat No. 70236, Merck, Germany). Sodium phosphate monobasic and dibasic (Cat No. 567545 and 567550), antibiotics (Cat No. 15140122, ThermoFisher Scientific, USA), DNAse (Cat No. 90083, ThermoFisher Scientific, USA), DMEM media (Cat No. D6429, Merck, Germany), PMSF (Cat No. 52332), xanthine (Cat No. X0626, Sigma, USA) 9-deazaguanine (Cat No. CS-0079471, Ambeed, USA), IMP (Cat No. I2879, Sigma, USA), PRPP (Cat No. P8296, Sigma, USA), IPTG (Cat No. EI05931, Carbosynth Ltd., (UK). Glycerol (Cat No. GB0232, Biobasic, Canada), LB broth (Cat No. SD7002, Biobasic, Canada), lysozyme (Cat No. LDB0308, Biobasic, Canada), TCEP (Cat No. TB0974, Biobasic, Canada). FPLC columns (Cat No. 17524701, GE Healthcare, USA). Human fibroblast BJ cell line (Cat No. CRL-2522, ATCC, USA), while MTT (Cas No. 298-93-1, MP Biomedicals, France), trypsin EDTA (Cat No. 25200056, Gibco, ThermoFisher Scientific, USA) FBS (Cat No. A5670701, Gibco, ThermoFisher Scientific, USA), and trypan blue (Cat No. 15250061, ThermoFisher Scientific, USA). The plasmid HGXPRT was a gift from Prof. Vern L. Schramm (Addgene plasmid # 64201; http://n2t.net/addgene:64201; RRID: Addgene_64201), Department of Biochemistry, Albert Einstein College of Medicine, Bronx, New York, USA. Deuterated solvents for the biophysical studies were purchased from Armar Chemicals (Switzerland), and Cambridge Isotope Laboratories, Inc. (USA).

### General procedure for the synthesis of analogues 1–17

Paroxetine HCl (0.607 mmol) was dissolved in acetonitrile (10 mL), and triethylamine (Et_3_N) (1 eq., 0.368 mmol) was added to neutralize the hydrochloride salt of paroxetine. The reaction mixture was then reflux for 20 minutes under temperature-controlled conditions using electric thermometer, heating was provided by using an oil bath. Later, 1.0 eq. of corresponding phenyl isothiocyanate was added to the reaction mixture, followed by reflux for 18–20 hours at 85 °C. Reaction progress was monitored by thin-layer chromatography (TLC) using hexane-ethyl acetate (7:3) as eluent. Upon completion, solvent was evaporated and crude compounds were subjected to the gradient column chromatography by using hexane-ethyl acetate as an eluent [14]. Pure compounds were then characterized by using standard spectroscopic techniques, including mass, UV, IR, ^1^H-NMR, and ^13^C-NMR spectroscopy.

#### Spectral data for the synthesized compounds.

***(3S,4R)-3-((Benzo[d][1,3]dioxol-5-yloxy)methyl)-4-(4-fluorophenyl)-N-phenylpiperidine-1-carbothioamide* (1):** Dull white solid (91%); R_f_ = 0.75, m. p. 80−85 °C; IR v_max_ 3219, 1509, 1323, 1267, 1036, 1184 cm^-1^; UV **λ**_max_ 248, 212 nm; ^1^H-NMR (400 MHz, DMSO-*d*_*6*_) *δ* 9.35 (s, 1H, NH), 7.31, 7.28 (ovp, 6H, H-2′′′, H-3′′′, 5′′′, 6′′′, H- 2′′, 6′′), 7.13, 7.08 (ovp, 3H, H-4′′′, H- 3′′, 5′′), 6.72 (d, *J*_7′/6′_ = 8.4 Hz, 1H, H-7′), 6.46 (d, *J*_4′/6′_ = 2.4 Hz, 1H, H-4′), 6.19 (dd, *J*_6´/7′_ = 8.8 Hz, *J*_6´/4′_ = 2.8 Hz, 1H, H-6′), 5.90 (s, 2H, H-2′), 5.08 (d, *J*_2a/2b_ = 12.4 Hz, 1H, H-2a), 4.88 (d, *J*_6a/6b_ = 12.8 Hz, 1H, H-6a), 3.60 (dd, *J*_7a/3_ = 9.6 Hz, *J*_7a/7b_ = 2.4 Hz, 1H, H-7a), 3.52 (dd, *J*_7b/3_ = 10.0 Hz, *J*_7b/7a_ = 6.4 Hz, 1H, H-7b), 3.14 (d, *J*_6b/5_ = 7.2 Hz, 1H, H-6b), 3.09 (d, *J*_2b/3_ = 12.0 Hz, 1H, H-2b), 2.88 (td, *J*_4/5_ = 13.6 Hz, *J*_4/3_ = 5.2 Hz, 1H, H-4), 2.13 (br, 1H, H-3), 1.79, (m, 2H, H-5); ^13^C-NMR (125 MHz, DMSO-*d*_*6*_) *δ* 181.3 (C = S, 1C), 161.8 (1C, C-4′), 153.8 (1C, C-5′), 147.7 (1C, C-3′), 127.9 (2C, C-3′′′/ C5′′′), 139.4 (1C, C-1′′), 128.3 (1C, C-1′′′), 127.9 (2C, C-3′′′ and C-5′′′), 129.2 (2C, C-2′′, C-6′′), 125.1 (2C, C-2′′′ and C-6′′′), 124.1 (1C, C-4′′′), 115.2 (2C, C-3′′ and C-5′′), 107.9 (1C, C-7′), 105.8 (1C, C-6′), 100.9 (1C, C-2′), 97.9 (1C, C-4′), 51.3 (1C, C-2), 48.15 (1C, C-6), 43.1 (1C, C-4), 41.2 (1C, C-3), 33.3 (1C, C-5);ESI-MS *m/z* 465 [M + H]^+^; HRFAB-MS *m/z* 465.1671 [M + H]^+^ (C_26_H_26_O_3_N_2_F_1_S_1_ requires 465.1648).

***(3S,4R)-3-((Benzo[d][1,3]dioxol-5-yloxy)methyl)-N-(4-chlorophenyl)-4-(4-fluorophenyl)-piperidine-1-carbothioamide* (2):** White solid (46%); R_f_ = 0.42, m. p. 83−86 °C; IR v_max_ 3230, 2918, 1509, 13121, 1269, 1185, 1037 cm^-1^; UV **λ**_max_ 247, 214 nm; ^1^H-NMR (400 MHz, DMSO-*d*_*6*_) *δ* 9.40 (s, 1 H, N-H), 7.33 (ovp, 4 H, H-2′′′, H-3′′′, H-5′′′, H-6′′′), 7.30 (dd, *J*_2´´/3′´_, _6´´/5′´_ = 8.4 Hz, *J*_2´´/4′´-F_, _6´´/4′´-F_ = 6.0 Hz, 2 H, H-2′′, H-6′′), 7.10 (t, *J*_3´´/2′´_, _3´´/4′´-F, 5´´/6′´_, _5´´/4′´-F_ = 8.8 Hz, 2 H, H-3′′, H-5′′), 6.72 (d, *J*_7′/6′_ = 8.4 Hz, 1 H, H-7′), 6.46 (d, *J*_4′/6′_ = 2.4 Hz, 1 H, H-4′), 6.20 (dd, *J*_6´/7′_ = 8.8 Hz, *J*_6´/4′_ = 2.8 Hz, 1 H, H-6′), 5.90 (s, 2 H, H-2′), 5.07 (d, *J*_2a/2b_ = 12.0 Hz, 1 H, H-2a), 4.87 (d, *J*_6a/6b_ = 12.8 Hz, 1 H, H-6a), 3.59 (dd, *J*_7a/3_ = 9.6 Hz, *J*_7a/7b_ = 2.4 Hz, 1 H, H-7a), 3.52 (dd, *J*_7b/3_ = 9.6 Hz, *J*_7b/7a_ = 6.4 Hz, 1 H, H-7b), 3.16 (d, *J*_6b/5_ = 8.0 Hz, 1 H, H-6b), 3.11 (d, *J*_2b/3_ = 11.6 Hz, 1H, H-2b), 2.88 (td, *J*_4/5_ = 10.8 Hz, *J*_4/3_ = 4.4 Hz, 1 H, H-4), 2.13 (br s, 1 H, H-3), 1.77 (m, 2 H, H-5); ^13^C-NMR (125 MHz, DMSO-*d*_*6*_) *δ* 181.1 (C = S, 1C), 161.5 (1C, C-4′), 153.8 (1C, C-5′), 147.8 (1C, C-3′), 127.8 (1C, C-3′′′), 141.2 (1C, C-1′′), 139.4 (1C, C-1′′), 129.3 (1C, C-1′′′), 127.8 (2C, C-3′′′/C-5′′′), 129.2 (2C, C-2′′ and C-6′′), 126.7 (2C, C-2′′′ and C-6′′′), 140.1 (1C, C-4′′′), 115.2 (2C, C-3′′ and C-5′′), 107.9 (1C, C-7′), 105.8 (1C, C-6′), 100.9 (1C, C-2′), 97.9 (1C, C-4′), 51.3 (1C, C-2), 48.6 (1C, C-6), 43.0 (1C, C-4), 41.2 (1C, C-3), 33.2 (1C, C-5); ESI-MS *m/z* 537 [M + Na]^+^, 501 [M + 2], 499 [M + H]^+^; HRFAB-MS *m/z* 499.1279 [M + H]^+^ (C_26_H_25_O_3_N_2_Cl_1_F_1_S_1_ requires 499.1258).

***(3S,4R)-3-((Benzo[d][1,3]dioxol-5-yloxy)methyl)-N-(3-chlorophenyl)-4-(4-fluorophenyl)piperidine-1-carbothioamide* (3):** White solid (61%), R_f_ = 0.48, m. p. 98−100 °C; IR v_max_ 3214, 2916, 1593, 1319, 1269, 1184, 1037 cm^-1^; UV **λ**_max_ 259, 248, 229, 219 nm; ^1^H-NMR (400 MHz, DMSO-*d*_*6*_) *δ* 9.41 (s, 1 H, N-H), 7.41 (s, 1 H, H-2′′′), 7.30–7.24 (ovp, 4 H, H-5′′′, H- 6′′′, H- 2′′, H-6′′), 7.11–7.06 (ovp, 3 H, H-4′′′, H- 3′′, H-5′′), 6.69 (d, *J*_7′/6′_ = 8.4 Hz, 1 H, H-7′), 6.44 (d, *J*_4′/6′_ = 2.4 Hz, 1 H, H-4′), 6.17 (dd, *J*_6´/7′_ = 8.8 Hz, *J*_6´/4′_ = 2.8 Hz, 1 H, H-6′), 5.88 (s, 2 H, H-2′), 5.03 (d, *J*_2a/2b_ = 12.4 Hz, 1 H, H-2a), 4.84 (d, *J*_6a/6b_ = 12.0 Hz, 1 H, H-6a), 3.58 (dd, *J*_7a/3_ = 10 Hz, *J*_7a/7b_ = 2.4 Hz, 1 H, H-7a), 3.50 (dd, *J*_7b/3_ = 9.6 Hz, *J*_7b/7a_ = 6.4 Hz, 1 H, H-7b), 3.13 (d, *J*_6b/5_ = 7.2 Hz, 1 H, H-6b), 3.09 (d, *J*_2b/3_ = 11.6 Hz, 1 H, H-2b), 2.87 (td, *J*_4/5_ = 10.8 Hz, *J*_4/3_ = 4.8 Hz, 1 H, H-4), 2.11 (br s, 1 H, H-3), 1.77 (m, 2 H, H-5); ^13^C-NMR (100 MHz, DMSO-*d*_*6*_) *δ* 180.9 (C = S, 1C), 161.8 (1C, C-4′), 153.8 (1C, C-5′), 147.7 (1C, C-3′), 142.7 (1C, C-3′′′), 141.2 (1C, C-1′′), 139.4 (1C, C-1′′), 132.0 (1C, C-1′′′), 129.4 (1C, C-5′′′), 129.2 (2C, C-2′′, C-6′′), 124.4 (1C, C-2′′′), 123.6 (1C, C-4′′′), 123.2 (1C, C-6′′′), 115.2 (2C, C-3′′ and C-5′′), 107.9 (1C, C-7′), 105.8 (1C, C-6′), 100.9 (1C, C-2′), 97.9 (1C, C-4′), 51.3 (1C, C-2), 48.7 (1C, C-6), 43.0 (1C, C-4), 41.2 (1C, C-3), 33.2 (1C, C-5); (ESI-MS *m/z* 501 [M + 2], 499 [M + H]^+^; HRFAB-MS *m/z* 499.1283 [M + H]^+^ (C_26_H_25_O_3_N_2_Cl_1_F_1_S_1_ requires 499.1258).

***(3S,4R)-3-((Benzo[d][1,3]dioxol-5-yloxy)methyl)-N-(3,4-dichlorophenyl)-4-(4-fluorophenyl)piperidine-1-carbothioamide* (4):** White solid (62%); R_f_ = 0.42, m. p. 69−74 °C; IR v_max_ 3246, 2918, 1509, 1315, 1220, 1184, 1037 cm^-1^; UV **λ**_max_ 248, 213 nm; ^1^H-NMR (400 MHz, DMSO-*d*_*6*_) *δ* 9.50 (s, 1H, NH), *δ* 7.64 (s, 1H, H-2′′′), 7.54 (d, *J*_6´´´/5′´´_ = 8.4 Hz, 1H, H-6′′′), 7.34–7.28 (ovp, 3H, H- 2′′, H-6′′, H-5′′′), 7.11 (t, *J*_3´´/2′´_, _3´´/4′´-F, 5´´/6′´_, _5´´/4′´-F_ = 8.4 Hz, 2H, H-3′′, 5′′), 6.72 (d, *J*_7′/6′_ = 8.4 Hz, 1H, H-7′), 6.47 (d, *J*_4′/6′_ = 2.4 Hz, 1H, H-4′), 6.20 (dd, *J*_6´/7′_ = 8.8 Hz, *J*_6´/4′_ = 2.8 Hz, 1H, H-6′), 5.90 (s, 2H, H-2′), 5.06 (d, *J*_2a/2b_ = 11.2 Hz, 1H, H-2a), 4.86 (d, *J*_6a/6b_ = 11.6 Hz, 1H, H-6a), 3.61 (d, *J*_7a/3_ = 8.0 Hz, 1H, H-7a), 3.52 (dd, *J*_7b/3_ = 15.2 Hz, *J*_7b/7a_ = 6.0 Hz, 1H, H-7b), 3.16 (d, *J*_6b/5_ = 8.4 Hz, 1H, H-6b), 3.14 (d, *J*_2b/3_ = 12.4 Hz, 1H, H-2b), 2.89 (td, *J*_4/5_ = 11.2 Hz, *J*_4/3_ = 6.8 Hz, 1H, H-4), 2.14 (br, 1H, H-3), 1.80 (m, 2H, H-5); ESI-MS *m/z* 537 [M + 4]^+^, 535 [M + 2]^+^, 533 [M + H]^+^; HRFAB-MS *m/z* 533.0889 [M + H]^+^ (C_26_H_24_O_3_N_2_Cl_2_F_1_S_1_ requires 533.0869).

***(3S,4R)-3-((Benzo[d][1,3]dioxol-5-yloxy)methyl)-N-(2,5-dichlorophenyl)-4-(4-fluorophenyl)piperidine-1-carbothioamide* (5):** Yellow solid (96%); R_f_ = 0.61, m. p. 62−66 °C; IR v_max_ 3238, 3238, 2918, 1509, 1317, 1270, 1184, 1037, cm^-1^; UV **λ**_max_ 227 nm; ^1^H-NMR (400 MHz, DMSO-*d*_*6*_) *δ* 9.33 (s, 1H, NH), 7.50 (d, *J*_3´´´/4′´´_ = 8.8 Hz, 1H, H-3′′′), 7.43 (d, *J*_6´´´/4′´´_ = 2.0 Hz, 1H, H-6′′′), 7.34 (dd, *J*_4´´´/3′´´_ = 8.8 Hz, *J*_4´´´/6′´´_ = 2.8 Hz, 1H, H-4′′′), 7.30 (dd, *J*_2´´/3′´_, _6´´/5′´_ = 8.4 Hz, *J*_2´´/4′´-F_, _6´´/4′´-F_ = 5.6 Hz, 2H, H-2′′, 6′′), 7.12 (t, *J*_3´´/2′´_, _3´´/4′´-F,_ *J*_5´´/6′´_, _5´´/4′´-F,_ = 8.8 Hz, 2H, H-3′′, 5′′), 6.72 (d, *J*_7′/6′_ = 8.4 Hz, 1H, H-7′), 6.47 (d, *J*_4′/6′_ = 2.4 Hz, 1H, H-4′), 6.20 (dd, *J*_6´/7′_ = 8.8 Hz, *J*_6´/4′_ = 2.8 Hz, 1H, H-6′), 5.90 (s, 2H, H-2′), 5.05 (d, *J*_2a/2b_ = 10.4 Hz, 1H, H-2a), 4.85 (d, *J*_6a/6b_ = 10.8 Hz, 1H, H-6a), 3.61 (dd, *J*_7a/3_ = 9.6 Hz, *J*_7a/7b_ = 2.4 Hz, 1H, H-7a), 3.53 (dd, *J*_7b/3_ = 9.6 Hz, *J*_7b/7a_ = 6.0 Hz, 1H, H-7b), 3.22 (d, *J*_6b/5_ = 10.0 Hz, 1H, H-6b), 3.16 (d, *J*_2b/3_ = 12.4 Hz, 1H, H-2b), 2.91 (td, *J*_4/5_ = 11.2 Hz, *J*_4/3_ = 4.8 Hz, 1H, H-4), 2.14 (br s, 1H, H-3), 1.75 (m, 2H, H-5); ESI-MS *m/z* 537 [M + 4]^+^, 535 [M + 2]^+^, 533 [M + H]^+^; HRFAB-MS *m/z* 533.0894 [M + H]^+^ (C_26_H_24_O_3_N_2_Cl_2_F_1_S_1_ requires 533.0869).

***(3S,4R)-3-((Benzo[d][1,3]dioxol-5-yloxy)methyl)-N-(3-bromophenyl)-4-(4-fluorophenyl)piperidine-1-carbothioamide* (6):** White solid (69%), R_f_ = 0.42, m. p. 80−84 °C; IR v_max_ 3227, 2917, 1587, 1319, 1269, 1184, 1037, cm^-1^; UV **λ**_max_ 243, 229, 213 nm; ^1^H-NMR (400 MHz, DMSO-*d*_*6*_) *δ* 9.43 (s, 1 H, N-H), 7.57 (s, 1 H, H-2′′′), 7.34–7.24 (ovp, 5H, H- 2′′, H-6′′, H-4′′′, H- 5′′′, H- 6′′′), 7.11 (t, *J*_3´´/2′´_, _3´´/4′´-F, 5´´/6′´_, _5´´/4′´-F_ = 8.8 Hz, 2 H, H-3′′, H-5′′), 6.72 (d, *J*_7′/6′_ = 8.4 Hz, 1 H, H-7′), 6.47 (d, *J*_4′/6′_ = 2.4 Hz, 1 H, H-4′), 6.20 (dd, *J*_6´/7′_ = 8.8 Hz, *J*_6´/4′_ = 2.8 Hz, 1 H, H-6′), 5.90 (s, 2H, H-2′), 5.06 (d, *J*_2a/2b_ = 11.6 Hz, 1 H, H-2a), 4.86 (d, *J*_6a/6b_ = 12.4 Hz, 1 H, H-6a), 3.61 (dd, *J*_7a/3_ = 9.6 Hz, *J*_7a/7b_ = 2.4 Hz, 1 H, H-7a), 3.52 (dd, *J*_7b/3_ = 10.0 Hz, *J*_7b/7a_ = 6.4 Hz, 1 H, H-7b), 3.17 (d, *J*_6b/5_ = 7.2 Hz, 1 H, H-6b), 3.12 (d, *J*_2b/3_ = 11.2 Hz, 1 H, H-2b), 2.89 (td, *J*_4/5_ = 11.2 Hz, *J*_4/3_ = 5.2 Hz, 1H, H-4), 2.14 (br s, 1H, H-3), 1.80 (m, 2H, H-5); ESI-MS *m/z* 545 [M + 2]^+^, 543 [M + H]^+^; HRFAB-MS *m/z* 543.0781 [M + H]^+^ (C_26_H_25_O_3_N_2_Br_1_F_1_S_1_ requires 543.0753).

***(3S,4R)-3-((Benzo[d][1,3]dioxol-5-yloxy)methyl)-N-(3-fluorophenyl)-4-(4-fluorophenyl)piperidine-1-carbothioamide* (7):** White solid (72%), R_f_ = 0.6, m. p. 73−76 °C; IR v_max_ 3233, 2918, 1509, 1321, 1271, 1184, 1037 cm^-1^; UV **λ**_max_ 260, 247 nm; ^1^H-NMR (400 MHz, DMSO-*d*_*6*_) *δ* 9.46 (s, 1 H, N-H), 7.30, 7.28 (ovp, 3H, H-2′′′, H- 2′′, H-6′′), 7.24 (dt, *J*_6´´´/5′´´_ = 4.0 Hz, *J*_6´´´/2′´´,6´´´/4′´´_*,*_6´´´/3′´′-F_ = 2.0 Hz, 1 H, H-6′′′), 7.15, 7.09 (ovp, 3H, H-5′′′, H- 3′′, H-5′′), 6.90 (td, *J*_4´´´/5′´´_*,*_4´´´/3′´′-F_ = 10.8 Hz, *J*_4´´´/2′´´, 4´´´/6′´´_ = 2.4 Hz, 1 H, H-4′′′), 6.72 (d, *J*_7′/6′_ = 8.4 Hz, 1 H, H-7′), 6.47 (d, *J*_4′/6′_ = 2.4 Hz, 1 H, H-4′), 6.19 (dd, *J*_6´/7′_ = 8.8 Hz, *J*_6´/4′_ = 2.8 Hz, 1 H, H-6′), 5.90 (s, 2 H, H-2′), 5.06 (d, *J*_2a/2b_ = 12.4 Hz, 1 H, H-2a), 4.86 (d, *J*_6a/6b_ = 12.8 Hz, 1 H, H-6a), 3.61 (dd, *J*_7a/3_ = 10.0 Hz, *J*_7a/7b_ = 2.4 Hz, 1 H, H-7a), 3.54 (dd, *J*_7b/3_ = 10.0 Hz, *J*_7b/7a_ = 6.4 Hz, 1 H, H-7b), 3.15 (d, *J*_6b/5_ = 8.0 Hz, 1H, H-6b), 3.12 (d, *J*_2b/3_ = 12.0 Hz, 1H, H-2b), 2.89 (td, *J*_4/5_ = 10.8 Hz, *J*_4/3_ = 4.8 Hz, 1H, H-4), 2.14 (br, 1H, H-3), 1.78 (m, 2H, H-5); ESI-MS *m/z* 483 [M + H]^+^; HRFAB-MS *m/z* 483.1533 [M + H]^+^ (C_26_H_25_O_3_N_2_F_2_S_1_ requires 483.1554).

***(3S,4R)-3-((Benzo[d][1,3]dioxol-5-yloxy)methyl)-N-(2-fluorophenyl)-4-(4-fluorophenyl)piperidine-1-carbothioamide* (8):** White solid (70%), R_f_ = 0.6, m. p. 71−76°C; IR v_max_ 3214, 3041, 2918, 1509, 1322, 1267, 1184, 1036, cm^-1^; UV **λ**_max_ 241 nm; ^1^H-NMR (400 MHz, DMSO-*d*_*6*_) *δ* 9.17 (s, 1H, NH), 7.31–7.09 (ovp, 8H, H- 2′′, H- 3′′, H-5′′, H-6′′, H-3′′′, H-4′′′, H-5′′′, H-6′′′), 6.72 (d, *J*_7′/6′_ = 8.4 Hz, 1H, H-7′), 6.47 (d, *J*_4′/6′_ = 2.0 Hz, 1H, H-4′), 6.21 (dd, *J*_6´/7′_ = 8.4 Hz, *J*_6´/4′_ = 2.0 Hz, 1H, H-6′), 5.90 (s, 2H, H-2′), 5.09 (d, *J*_2a/2b_ = 12.0 Hz, 1H, H-2a), 4.88 (d, *J*_6a/6b_ = 11.6 Hz, 1H, H-6a), 3.61 (dd, *J*_7a/3_ = 7.6 Hz, *J*_7a/7b_ = 2.4 Hz, 1H, H-7a), 3.54 (dd, *J*_7b/3_ = 16.0 Hz, *J*_7b/7a_ = 6.0 Hz, 1H, H-7b), 3.19 (d, *J*_6b/5_ = 11.2 Hz, 1H, H-6b), 3.16 (d, *J*_2b/3_ = 12.4 Hz, 1H, H-2b), 2.88 (td, *J*_4/5_ = 11.2 Hz, *J*_4/3_ = 7.2 Hz, 1H, H-4), 2.13 (br, 1H, H-3), 1.77 (m, 2H, H-5); ESI-MS *m/z* 483 [M + H]^+^; HRFAB-MS *m/z* 483.1531 [M + H]^+^ (C_26_H_25_O_3_N_2_F_2_S_1_ requires 483.1554).

***(3S,4R)-3-((Benzo[d][1,3]dioxol-5-yloxy)methyl)-N,4-bis(4-fluorophenyl)-piperidine-1-carbothioamide* (9):** White solid (65%), R_f_ = 0.35, m. p. 78−82°C; IR v_max_ 3230, 2918, 1509, 1325, 1269, 1184,1037 cm^-1^; UV **λ**_max_ 243 nm; ^1^H-NMR (400 MHz, DMSO-*d*_*6*_) *δ* 9.33 (s, 1H, NH), 7.31–7.28 (ovp, 4H, H- 2′′, H-6′′, H-2′′′, H-6′′′), 7.14–7.08 (ovp, 4H, H- 3′′, H-5′′, H-3′′′, H-5′′′), 6.72 (d, *J*_7′/6′_ = 8.4 Hz, 1H, H-7′), 6.47 (d, *J*_4′/6′_ = 2.4 Hz, 1H, H-4′), 6.20 (dd, *J*_6´/7′_ = 8.8 Hz, *J*_6´/4′_ = 2.8 Hz, 1H, H-6′), 5.90 (s, 2H, H-2′), 5.09 (d, *J*_2a/2b_ = 12.8 Hz, 1H, H-2a), 4.89 (d, *J*_6a/6b_ = 12.8 Hz, 1H, H-6a), 3.61 (dd, *J*_7a/3_ = 10.0 Hz, *J*_7a/7b_ = 2.4 Hz, 1H, H-7a), 3.54 (dd, *J*_7b/3_ = 9.6 Hz, *J*_7b/7a_ = 6.0 Hz, 1H, H-7b), 3.13 (d, *J*_6b/5_ = 8.0 Hz, 1H, H-6b), 3.10 (d, *J*_2b/3_ = 11.6 Hz, 1H, H-2b), 2.89 (td, *J*_4/5_ = 11.2 Hz, *J*_4/3_ = 4.4 Hz, 1H, H-4), 2.12 (br s, 1H, H-3), 1.76 (m, 2H, H-5); ESI-MS *m/z* 483 [M + H]^+^; HRFAB-MS *m/z* 483.1578 [M + H]^+^ (C_26_H_25_O_3_N_2_F_2_S_1_ requires 483.1554).

***(3S,4R)-3-((Benzo[d][1,3]dioxol-5-yloxy)methyl)-N-(2,4-difluorophenyl)-4-(4-fluorophenyl)piperidine-1-carbothioamide* (10):** Light yellow solid (96%); R_f_ = 0.42, m. p. 77−79 °C; IR v_max_ 3228, 2918, 1510, 1324, 1266, 1185, 1037, cm^-1^; UV **λ**_max_ 230, 213 nm; ^1^H-NMR (400 MHz, DMSO-*d*_*6*_) *δ* 9.15 (s, 1H, NH), 7.31–7.22 (ovp, 4H, H- 2′′, H-6′′, H-5′′′, 6′′′),7.11 (t, *J*_3´´/2′´_, _3´´/4′´-F, 5´´/6′´_, _5´´/4′´-F_ = 8.8 Hz, 2H, H-3′′, H-5′′), 7.06 (td, *J*_3´´´/2′´′-F_, _3´´´/4′´′-F_ = 8.4 Hz, *J*_3´´´/ 5´´´_ = 1.6 Hz, 1H, H-2′′′), 6.72 (d, *J*_7′/6′_ = 8.4 Hz, 1H, H-7′), 6.47 (d, *J*_4′/6′_ = 2.4 Hz, 1H, H-4′), 6.21 (dd, *J*_6´/7′_ = 8.8 Hz, *J*_6´/4′_ = 2.8 Hz, 1H, H-6′), 5.90 (s, 2H, H-2′), 5.08 (d, *J*_2a/2b_ = 11.6 Hz, 1H, H-2a), 4.87 (d, *J*_6a/6b_ = 11.2 Hz, 1H, H-6a), 3.61 (dd, *J*_7a/3_ = 9.6 Hz, *J*_7a/7b_ = 2.4 Hz, 1H, H-7a), 3.53 (dd, *J*_7b/3_ = 9.6 Hz, *J*_7b/7a_ = 6.4 Hz, 1H, H-7b), 3.19 (d, *J*_6b/5_ = 10.8 Hz, 1H, H-6b), 3.13 (d, *J*_2b/3_ = 12.8 Hz, 1H, H-2b), 2.89 (td, *J*_4/5_ = 11.6 Hz, *J*_4/3_ = 4.0 Hz, 1H, H-4), 2.12 (br, 1H, H-3), 1.77 (m, 2H, H-5); ESI-MS *m/z* 501 [M + H]^+^; HRFAB-MS *m/z* 501.1488 [M + H]^+^ (C_26_H_24_O_3_N_2_F_3_S_1_ requires 501.1460).

***(3S,4R)-3-((Benzo[d][1,3]dioxol-5-yloxy)methyl)-N-(3,4-difluorophenyl)-4-(4-fluorophenyl)piperidine-1-carbothioamide* (11):** Light yellow solid (74%), R_f_ = 0.33, m. p. 68−72 °C; IR v_max_ 3247, 3047, 2919, 1510, 1323, 1268, 1184, 1037 cm^-1^; UV **λ**_max_ 242, 210 nm; ^1^H-NMR (400 MHz, DMSO-*d*_*6*_) *δ* 9.43 (s, 1H, NH), 7.43 (ddd, *J*_6´´´/5′´´_ = 10.0 Hz, *J*_6´´´/2′´´_ = 2.8 Hz, *J*_6´´´/4′´′-F_ = 5.2 Hz, 1H, H-6′′′), 7.36 (t, *J*_2´´´/3′´′-F_ = 9.2 Hz, 1H, H-2′′′), 7.30 (dd, *J*_2´´/3′´_, _6´´/5′´_ = 8.4 Hz, *J*_2´´/4′´-F_, _6´´/4′´-F_ = 5.6 Hz, 2H, H-2′′, H-6′′), 7.11 (t, *J*_5´´´/6′´´_, _5´´´/4′´′-F,_ *J*_3´´/2′´_, _3´´/4′´-F, 5´´/6′´_, _5´´/4′´-F_ = 8.8 Hz, 3H, H-3′′, H-5′′, H-5′′′), 6.72 (d, *J*_7′/6′_ = 8.4 Hz, 1H, H-7′), 6.47 (d, *J*_4′/6′_ = 2.4 Hz, 1H, H-4′), 6.20 (dd, *J*_6´/7′_ = 8.8 Hz, *J*_6´/4′_ = 2.8 Hz, 1H, H-6′), 5.90 (s, 2H, H-2′), 5.07 (d, *J*_2a/2b_ = 12.4 Hz, 1H, H-2a), 4.87 (d, *J*_6a/6b_ = 12.4 Hz, 1H, H-6a), 3.61 (dd, *J*_7a/3_ = 10.0 Hz, *J*_7a/7b_ = 2.8 Hz, 1H, H-7a), 3.53 (dd, *J*_7b/3_ = 10.0 Hz, *J*_7b/7a_ = 6.4 Hz, 1H, H-7b), 3.17 (d, *J*_6b/5_ = 8.0 Hz, 1H, H-6b), 3.12 (d, *J*_2b/3_ = 11.6 Hz, 1H, H-2b), 2.88 (td, *J*_4/5_ = 11.2 Hz, *J*_4/3_ = 6.8 Hz, 1H, H-4), 2.13 (br s, 1H, H-3), 1.77 (m, 2H, H-5); ESI-MS *m/z* 501 [M + H]^+^; HR FAB-MS *m/z* 501.1484 [M + H]^+^ (C_26_H_24_O_3_N_2_F_3_S_1_ requires 501.1460).

***(3S,4R)-3-((Benzo[d][1,3]dioxol-5-yloxy)methyl)-4-(4-fluorophenyl)-N-(3-nitrophenyl) piperidine-1-carbothioamide* (12):** Yellow solid (68%), R_f_ = 0.26, m. p. 99−101 °C; IR v_max_ 3213, 1529, 1347, 1509, 1316, 1268, 1036, 1184 cm^-1^; UV **λ**_max_ 250, 212 nm; ^1^H-NMR (400 MHz, DMSO-*d*_*6*_) *δ* 9.70 (s, 1 H, N-H), 8.28 (d, *J*_2´´´/4′´´_, _2´´´/6′´´_ = 2.0 Hz, 1 H, H-2′′′), 7.93 (dd, *J*_4´´´/5′´´_ = 8.0 Hz, *J*_4´´´/2′´´_, _4´´´/6′´´_ = 1.6 Hz, 1 H, H-4′′′), 7.82 (d, *J*_6´´´/5′´´_ = 8.4 Hz, 1 H, H-6′′′), 7.56 (t, *J*_5´´´/4′´´, 5´´´/6′´´_ = 8.4 Hz, 1 H, H-5′′′), 7.32 (dd, *J*_2´´/3′´_, _6´´/5′´_ = 8.4 Hz, *J*_2´´/4′´-F_, _6´´/4′´-F_ = 5.6 Hz, 2 H, H-2′′, H-6′′), 7.11 (t, *J*_3´´/2′´_, _3´´/4′´-F, 5´´/6′´_, _5´´/4′´-F_ = 9.2 Hz, 2 H, H-3′′, H-5′′), 6.72 (d, *J*_7′/6′_ = 8.4 Hz, 1 H, H-7′), 6.47 (d, *J*_4′/6′_ = 2.4 Hz, 1 H, H-4′), 6.20 (dd, *J*_6´/7′_ = 8.8 Hz, *J*_6´/4′_ = 2.8 Hz, 1 H, H-6′), 5.90 (s, 2 H, H-2′), 5.10 (d, *J*_2a/2b_ = 12.0 Hz, 1 H, H-2a), 4.91 (d, *J*_6a/6b_ = 11.6 Hz, 1 H, H-6a), 3.62 (dd, *J*_7a/3_ = 9.6 Hz, *J*_7a/7b_ = 2.4 Hz, 1 H, H-7a), 3.53 (dd, *J*_7b/3_ = 10 Hz, *J*_7b/7a_ = 6.4 Hz, 1 H, H-7b), 3.21 (d, *J*_6b/5_ = 6.0 Hz, 1 H, H-6b), 3.17 (d, *J*_2b/3_ = 11.2 Hz, 1 H, H-2b), 2.91 (td, *J*_4/5_ = 11.2 Hz, *J*_4/3_ = 5.2 Hz, 1H, H-4), 2.18 (br, 1 H, H-3), 1.20 (m, 2 H, H-5); ESI-MS *m/z* 510 [M + H]^+^; HRFAB-MS *m/z* 510.1475 [M + H]^+^ (C_26_H_25_O_5_N_3_ F_1_S_1_ requires 510.1499).

***(3S,4R)-3-((Benzo[d][1,3]dioxol-5-yloxy)methyl)-4-(4-fluorophenyl)-N-4-nitrophenyl) piperidine-1-carbothioamide* (13):** Yellow solid (89%); R_f_ = 0.54, m. p. 150−153°C; IR v_max_ 3301, 1540, 1341, 1508, 1288, 1267, 1035, 1184 cm^-1^; UV **λ**_max_ 296, 229 nm; ^1^H-NMR (400 MHz, DMSO-*d*_*6*_) *δ* 9.87 (s, 1 H, N-H), 8.17 (dd, *J*_3´´´/2′´´_, _5´´´/6′´´_ = 8.0 Hz, *J*_3´´´/5′´´_, _5´´´/3′´´_ = 2.0 Hz, 2 H, H-3′′′, H-5′′′), 7.61 (dd, *J*_2´´´/3′´´_, _6´´´/5′´´_ = 8.0 Hz, *J*_2´´´/6′´´_, _6´´´/2′´´_ = 2.0 Hz, 2 H, H-2′′′, H-6′′′), 7.31 (dd, *J*_2´´/3′´_, _6´´/5′´_ = 8.4 Hz, *J*_2´´/4′´-F_, _6´´/4′´-F_ = 5.6 Hz, 2 H, H-2′′, H-6′′), 7.11 (t, *J*_3´´/2′´_, _3´´/4′´-F, 5´´/6′´_, _5´´/4′´-F_ = 8.8 Hz, 2 H, H-3′′, H-5′′), 6.71 (d, *J*_7′/6′_ = 8.4 Hz, 1 H, H-7′), 6.45 (d, *J*_4′/6′_ = 2.4 Hz, 1 H, H-4′), 6.18 (dd, *J*_6´/7′_ = 8.8 Hz, *J*_6´/4′_ = 2.8 Hz, 1 H, H-6′), 5.90 (s, 2 H, H-2′), 5.03 (d, *J*_2a/2b_ = 10.4 Hz, 1 H, H-2a), 4.84 (d, *J*_6a/6b_ = 8.4 Hz, 1H, H-6a), 3.59 (dd, *J*_7a/3_ = 10.0 Hz, *J*_7a/7b_ = 2.8 Hz, 1H, H-7a), 3.53 (dd, *J*_7b/3_ = 10.0 Hz, *J*_7b/7a_ = 6.4 Hz, 1H, H-7b), 3.18 (d, *J*_6b/5_ = 12.1 Hz, 1H, H-6b), 3.15 (d, *J*_2b/3_ = 5.2 Hz, 1H, H-2b), 2.91 (td, *J*_4/5_ = 11.2 Hz, *J*_4/3_ = 4.8 Hz, 1H, H-4), 2.18 (br, 1H, H-3), 1.83 (m, 2H, H-5); ^13^C-NMR (125 MHz, DMSO-*d*_*6*_) *δ* 180.6 (C = S, 1C), 161.8 (1C, C-4′), 153.8 (1C, C-5′), 147.9 (1C, C-3′), 124 (2C, C-3′′′/ C5′′′), 139.3 (1C, C-1′′), 148 (1C, C-1′′′), 124 (2C, C-3′′′ and C-5′′′), 129.2 (2C, C-2′′, C-6′′), 122.2 (2C, C-2′′′ and C-6′′′), 141.9 (1C, C-4′′′), 115.2 (2C, C-3′′ and C-5′′), 107.9 (1C, C-7′), 105.8 (1C, C-6′), 101 (1C, C-2′), 97.8 (1C, C-4′), 51.7 (1C, C-2), 49.1 (1C, C-6), 42.9 (1C, C-4), 41.3 (1C, C-3), 33.3 (1C, C-5);ESI-MS *m/z* 510 [M + H]^+^; HRFAB-MS *m/z* 510.1519 [M + H]^+^ (C_26_H_25_O_5_N_3_F_1_S_1_ requires 510.1499).

***(3S,4R)-3-((Benzo[d][1,3]dioxol-5-yloxy)methyl)-4-(4-fluorophenyl)-N-(4-(trifluoromethoxy)phenyl)piperidine-1-carbothioamide* (14):** Dull white solid (72%); R_f_ = 0.48, m. p. 66−70 °C; IR v_max_ 3245, 1509, 1285, 1324, 1258, 1037, 1184 cm^-1^; UV **λ**_max_ 243, 213 nm; ^1^H-NMR (400 MHz, DMSO-*d*_*6*_) *δ* 9.45 (s, 1H, NH), 7.43 (d, *J*_3´´´/2′´´, 5´´´/6′´´_ = 8.8 Hz, 2H, H-3′′′, 5′′′), 7.32, 7.27 (ovp, 4H, H-5′′′, 6′′′, H- 2′′, 6′′), 7.11 (t, *J*_3´´/2′´_, _3´´/4′´-F,_ *J*_5´´/6′´_, _5´´/4′´-F,_ = 8.8 Hz, 2H, H-3′′, 5′′), 6.72 (d, *J*_7′/6′_ = 8.4 Hz, 1H, H-7′), 6.47 (d, *J*_4′/6′_ = 2.4 Hz, 1H, H-4′), 6.20 (dd, *J*_6´/7′_ = 8.8 Hz, *J*_6´/4′_ = 2.8 Hz, 1H, H-6′), 5.90 (s, 2H, H-2′), 5.09 (d, *J*_2a/2b_ = 12.4 Hz, 1H, H-2a), 4.88 (d, *J*_6a/6b_ = 12.8 Hz, 1H, H-6a), 3.61 (dd, *J*_7a/3_ = 10.0 Hz, *J*_7a/7b_ = 2.8 Hz, 1H, H-7a), 3.53 (dd, *J*_7b/3_ = 10.0 Hz, *J*_7b/7a_ = 6.4 Hz, 1H, H-7b), 3.17(d, *J*_6b/5_ = 7.6 Hz, 1H, H-6b), 3.12 (d, *J*_2b/3_ = 11.6 Hz, 1H, H-2b), 2.89 (td, *J*_4/5_ = 10.8 Hz, *J*_4/3_ = 6.0 Hz, 1H, H-4), 2.14 (br, 1H, H-3), 1.78 (m, 2H, H-5); HRFAB-MS *m/z* 549.1499 [M + H]^+^ (C_27_H_25_O_4_N_2_F_4_S_1_ requires 549.1471).

***(3S,4R)-3-((Benzo[d][1,3]dioxol-5-yloxy)methyl)-4-(4-fluorophenyl)-N-(4-methoxyphenyl)piperidine-1-carbothioamide* (15):** White solid (50%); R_f_ = 0.35, m. p. 87−89 °C; IR v_max_ 3232, 1509, 1328, 1243, 1035, 1182 cm^-1^; UV **λ**_max_ 247, 212 nm; ^1^H-NMR (400 MHz, DMSO-*d*_*6*_) *δ* 9.22 (s, s1H, NH), 7.30 (dd, *J*_2´´/3′´_, _6´´/5′´_ = 8.4 Hz, *J*_2´´/4′´-F_, _6´´/4′´-F_ = 5.6 Hz, 2H, H-2′′, 6′′), 7.17 (d, *J*_2´´´/3′´´, 6´´´/5′´´_ = 8.8 Hz, 2H, H-2′′′, 6′′′), 7.10 (t, *J*_3´´/2′′_, _3´´/4′´-F,_ *J*_5´´/6′´_, _5´´/4′´-F,_ = 8.8 Hz, 2H, H-3′′, 5′′), 6.87 (d, *J*_3´´´/2′´´, 5´´´/6′´´_ = 8.8 Hz, 2H, H-3′′′, 5′′′),6.72 (d, *J*_7′/6′_ = 8.4 Hz, 1H, H-7′), 6.47 (d, *J*_4′/6′_ = 2.4 Hz, 1H, H-4′), 6.20 (dd, *J*_6´/7′_ = 8.8 Hz, *J*_6´/4′_ = 2.8 Hz, 1H, H-6′), 5.90 (s, 2H, H-2′), 5.09 (d, *J*_2a/2b_ = 12.4 Hz, 1H, H-2a), 4.88 (d, *J*_6a/6b_ = 12.4 Hz, 1H, H-6a), *δ* 3.73 (s, 3H, OCH_3_), 3.60 (dd, *J*_7a/3_ = 7.2 Hz, *J*_7a/7b_ = 2.4 Hz, 1H, H-7a), 3.51 (dd, *J*_7b/3_ = 10.0 Hz, *J*_7b/7a_ = 6.4 Hz, 1H, H-7b), 3.14 (d, *J*_6b/5_ = 8.4 Hz, 1H, H-6b), 3.07 (d, *J*_2b/3_ = 11.6 Hz, 1H, H-2b), 2.88 (td, *J*_4/5_ = 11.2 Hz, *J*_4/3_ = 4.8 Hz, 1H, H-4), 2.13 (br, 1H, H-3), 1.79, (m, 2H, H-5); ESI-MS *m/z* 495 [M + H]^+^; HR FAB-MS *m/z* 495.1777 [M + H]^+^ (C_27_H_28_O_4_N_2_F_1_S_1_ requires 495.1754).

***(3S,4R)-3-((Benzo[d][1,3]dioxol-5-yloxy)methyl)-4-(4-fluorophenyl)-N-(3-(trifluoromethyl)phenyl)piperidine-1-carbothioamide* (16):** Light yellow solid (75%); R_f_ = 0.42, m. p. 68−70 °C; IR v_max_ 3253, 2919, 1510, 1331, 1269, 1184, 1037 cm^-1^; UV **λ**_max_ 247, 213 nm; ^1^H-NMR (400 MHz, DMSO-*d*_*6*_) *δ* 9.56 (s, 1H, NH), *δ* 7.70 (s, 1H, H-2′′′), 7.66 (d, *J*_4´´´/5′´´_ = 8.0 Hz, 1H, H-4′′′), 7.52 (t, *J*_5´´´/4′´´_, _5´´´/6′´´_ = 7.6 Hz, 1H, H-5′′′), 7.43 (d, *J*_4´´´/5′´´_ = 7.6 Hz, 1H, H-6′′′), 7.32 (dd, *J*_2´´/3′´_, _6´´/5′´_ = 8.0 Hz, *J*_2´´/4′´-F_, _6´´/4′´-F_ = 5.6 Hz, 2H, H-2′′, H-6′′), 7.11 (t, *J*_3´´/2′´_, _3´´/4′´-F, 5´´/6′´_, _5´´/4′´-F_ = 8.4 Hz, 2H, H-3′′, H-5′′), 6.72 (d, *J*_7′/6′_ = 8.4 Hz, 1H, H-7′), 6.47 (d, *J*_4′/6′_ = 2.4 Hz, 1H, H-4′), 6.20 (dd, *J*_6´/7′_ = 8.8 Hz, *J*_6´/4′_ = 2.8 Hz, 1H, H-6′), 5.90 (s, 2H, H-2′), 5.09 (d, *J*_2a/2b_ = 12.0 Hz, 1H, H-2a), 4.90 (d, *J*_6a/6b_ = 12.8 Hz, 1H, H-6a), 3.60 (dd, *J*_7a/3_ = 9.6 Hz, *J*_7a/7b_ = 7.6 Hz, 1H, H-7a), 3.53 (dd, *J*_7b/3_ = 9.6 Hz, *J*_7b/7a_ = 6.0 Hz, 1H, H-7b), 3.19 (d, *J*_6b/5_ = 6.8 Hz, 1H, H-6b), 3.16 (d, *J*_2b/3_ = 12.0 Hz, 1H, H-2b), 2.91 (td, *J*_4/5_ = 10.8 Hz, *J*_4/3_ = 5.2 Hz, 1H, H-4), 2.15 (br s, 1H, H-3), 1.76 (m, 2H, H-5); ESI-MS *m/z* 533 [M + H]^+^; HR FAB-MS *m/z* 533.1499 [M + H]^+^ (C_27_H_25_O_3_N_2_F_4_S_1_ requires 533.1522).

***(3S,4R)-3-((Benzo[d][1,3]dioxol-5-yloxy)methyl)-N-(4-cyanophenyl)-4-(4-fluorophenyl)piperidine-1-carbothioamide* (17):** Light yellow solid (91%); R_f_ = 0.51, m. p. 91−95 °C; IR v_max_ 3297, 2918, 2223, 1509, 1302, 1266, 1184, 1037 cm^-1^; UV **λ**_max_ 272, 219 nm; ^1^H-NMR (400 MHz, DMSO-*d*_*6*_) *δ* 9.69 (s, 1H, NH), 7.73 (d, *J*_3´´´/2′´´_, _5´´´/6′´´_ = 8.8 Hz, 2H, H-3′′′, H-5′′′), 7.55 (d, *J*_2´´´/3′´´_, _6´´´/5′´´_ = 8.8 Hz, 2H, H-2′′′, H-6′′′), 7.30 (dd, *J*_2´´/3′´_, _6´´/5′´_ = 8.4 Hz, *J*_2´´/4′´-F_, _6´´/4′´-F_ = 5.6 Hz, 2H, H-2′′, H-6′′), 7.11 (t, *J*_3´´/2′´_, _3´´/4′´-F, 5´´/6′´_, _5´´/4′´-F_ = 9.2 Hz, 2H, H-3′′, H-5′′), 6.72 (d, *J*_7′/6′_ = 8.4 Hz, 1H, H-7′), 6.45 (d, *J*_4′/6′_ = 2.4 Hz, 1H, H-4′), 6.19 (dd, *J*_6´/7′_ = 8.8 Hz, *J*_6´/4′_ = 2.8 Hz, 1H, H-6′), 5.90 (s, 2H, H-2′), 5.04 (d, *J*_2a/2b_ = 12.0 Hz, 1H, H-2a), 4.84 (d, *J*_6a/6b_ = 12.4 Hz, 1H, H-6a), 3.61 (dd, *J*_7a/3_ = 10.0 Hz, *J*_7a/7b_ = 2.8 Hz, 1H, H-7a), 3.53 (dd, *J*_7b/3_ = 9.6 Hz, *J*_7b/7a_ = 6.0 Hz, 1H, H-7b), 3.21 (d, *J*_6b/5_ = 8.4 Hz, 1H, H-6b), 3.15 (d, *J*_2b/3_ = 11.6 Hz, 1H, H-2b), 2.90 (td, *J*_4/5_ = 10.8 Hz, *J*_4/3_ = 4.4 Hz, 1H, H-4), 2.16 (br s, 1H, H-3), 1.79 (m, 2H, H-5); ESI-MS *m/z* 490 [M + H]^+^; HR FAB-MS *m/z* 490.1579 [M + H]^+^ (C_27_H_25_O_3_N_3_F_1_S_1_ requires 490.1601).

#### Transformation, expression, and purification of HGXPRT enzyme.

Gene encoding the HGXPRT enzyme was integrated into pDEST14 vector with *N*- terminal His tag, followed by a thrombin protease cleavage site upstream of the HGXPRT coding sequence It was next transformed into in *E. coli* BL21(DE3) pLysS cells. Protein expression was induced by IPTG, and purified by using affinity, and desalting chromatography, as described previously by our group [11,12].

#### *In vitro* enzyme inhibition assay.

Method reported by Raman and Hazleton *et al.* was followed to evaluate the HGXPRT inhibitory activity [12,13] with some modifications [15]. Briefly, the reaction mixture (200 μL) consisted of IMP pre-incubated enzyme (0.75 µM/well, 5 μL), substrate PRPP (1 mM, 20 μL), and test compound (0.25 mM, 10 μL) in assay buffer, containing 100 mM Tris HCl (pH 7.5), 0.5 mM DTT, and 12 mM MgCl_2_. The mixture was incubated at 25 ºC for 10 minutes. After that, substrate xanthine (0.3 mM, 20 μL) was added to initiate the enzyme reaction. Product formation was recorded as change in rate of enzyme reaction kinetically after every minute at 255 nm, for 15 minutes in ELISA plate reader (Spectra Max-340, Molecular Devices, USA). The control reaction contained the enzyme and substrate with no test compound or inhibitor (standard). To ensure that the product formation is enzyme catalyzed, a blank was also run that contained substrate and buffer only. The test compounds were dissolved in dimethyl sulfoxide (DMSO) with a final 5% concentration in the reaction mixture. The activity of enzyme was initially checked in 1, 5, and 10% of the DMSO and the enzyme was found to be catalytically active till 5% of DMSO in final reaction volume. 9-Deazaguanine was used as a standard inhibitor. All reactions were performed in triplicate and independently repeated three times

The percent inhibition of enzyme activity was measured by using the following formula:


$$\begin{array}{c} PercentInhibition=\text{1}\text{0}\text{0}-(\frac{Rateofreactionofenzymeinthepresenceof\;testcompound}{Rateofreactionofenzymeofcontrol}\times \text{100}) \end{array}$$

For the IC_50_ value determination, percent inhibition of enzyme activity was determined at various concentrations (250–3.125 µM) of the test compounds.

#### Biophysical analysis by STD-NMR spectroscopy.

NMR experiments were performed on Avance NEO 800 MHz NMR spectrometer at 298 K, equipped with 5 mm TCI cryoprobe (Bruker, Switzerland). Compounds were initially prepared as 100 mM stock solutions in CDCl_3_, and subsequently diluted to 1 mM in buffer (10 mM phosphate, 20 mM NaCl) of pH 7.5. Samples were prepared in deuterated buffer with ligand to protein ratio of 100:1.

The structural integrity of compounds was confirmed by a ^1^H-NMR experiment (Bruker standard *zgesgp* pulse program). For STD-NMR experiment, *stdiffesgp.3* pulse sequence was employed [16–20], with on-, and off-resonance irradiation frequencies set at −134 and 18,000 Hz, respectively. For selective irradiation, a train of 40 Gaussian pulses with a field strength of 86 Hz was used; each pulse had a length of 50 ms and was separated by a 0.1 s delay. A relaxation delay (d1) of 3.1 s and saturation time (d20) of 3 seconds were used, followed by spin lock filter (d29) of 30 ms with field strength of 4,960 Hz to suppress residual protein signals. Each dataset was acquired with 16 dummy scans and 1024 scans. The amplification factor of the ligand’s proton was calculated by using the following formula:


$$\begin{array}{c} STDAmplificationFactor=\left(\frac{I_{std}}{I_{\text{0}}}\right)\times LigandExcess \end{array}$$

where *I*_*0*_ and *I*_*std*_ are intensities of the proton signal in the off-resonance, and difference NMR spectra, respectively.

#### *In silico* molecular docking.

*In silico* studies were performed on active compounds in order to analyse the enzyme-ligand interactions at the atomic level by using Glide 6.9 module of the Schrödinger suite of programs, version 2023–1. For this purpose, crystal structure of HGXPRT enzyme, complexed with its substrate, i.e., hypoxanthine (PDB ID: 3OZF), was used as receptor protein [21,22]. In brief, the protein was prepared, optimized, and minimized employing the default settings of protein preparation wizard tool and OPLS3 force field. Structures of ligands were drawn in ChemDraw Professional software. Ligands were subsequently prepared with the *LigPrep* tool. In our study, the catalytic-site-centered grid used previously could not accommodate the current ligand series due to distinct structural features [13]. Due to this reason, we have employed SiteMap analysis to identify five alternative binding pockets in 3OZF protein structure [13]. The suitable grid box of 10 × 10 × 10 Å was generated using a receptor grid generation tool. Protein and ligands were docked using Glide XP module, and results were interpreted by analysing the final docked pose, and docking scores of each ligand. Binding energies of the ligand-receptor complex were calculated *via* Prime MM/GBSA module which further validate the docked poses [13,23–30].

#### Molecular dynamics (MD) simulation.

Molecular dynamics (MD) simulations of Hypoxanthineguanine xanthine phosphoribosyl transferase (PDB ID: 3OZF) and ligand complexes were conducted using the Desmond module in the Schrödinger suite to assess the structural stability of chosen ligands in the binding sites. The glide dock file was brought into the maestro workspace. The system was configured by incorporating the target molecule into a specific solvent environment, using the SPC water model within an orthorhombic box, neutralized with counter ions at a 0.1 M salt concentration, and energy-minimized to replicate physiological conditions. Once the system was established, MD simulation parameters were configured. Parameters from the OPLS4 force field were utilized to precisely depict interatomic interactions. Structure optimized to eliminate steric clashes, enabling suitable conditions for molecular dynamics simulations under NPT ensemble conditions at 300 K and 1 atm to maintain both temperature and pressure [23–27]. Simulations lasted 200 ns, achieving convergence with stabilized structural parameters (RMSF, radius of gyration (Rg), hydrogen bond occupancy, and binding stability metrics) and consistent protein–ligand interactions, indicating equilibrium was reached. Trajectory data were examined to clarify conformational stability, intermolecular interactions, and essential dynamic properties, guaranteeing thorough sampling and reproducibility in line with standards in computational biophysics.

#### *In silico* ADMET analysis.

*In silico* ADMET profiling was performed using QikProp in the Maestro interface to predict the pharmacokinetic and drug-like properties of candidate compounds for lead optimization. Ligand structures were imported in SDF format, and pre-processed with LigPrep to ensure correct protonation, stereochemistry, and tautomerism before assessment. QikProp utilizes empirical and theoretical descriptors from quantitative structure-activity relationship models to predict parameters, such as solubility, permeability, metabolic stability, and toxicity. Employing Lipinski’s Rule of Five, this tool systematically assesses compounds based on key physicochemical parameters that influence oral bioavailability, thereby help in the early identification of candidates with a lower likelihood of failure during drug development.

#### Cytotoxicity studies.

Compounds identified active in *in vitro* biochemical assay were then evaluated for cytotoxicity by standard MTT assay. This assay is based on the reduction of MTT to formazan salt by mitochondrial succinate dehydrogenase enzyme which appears as needle-like (crystals). This is a standard method for assessing cell viability [31,32].

Briefly, the assay was performed on human fibroblast cells (BJ, CRL-2522). The cell line was obtained from the Biobank facility (PCMD, ICCBS), which was procured from ATCC, USA. The frozen vial of cells was thawed by gentle agitation in water bath at 37 °C for 2 min. The cells were aseptically transferred to 75 cc flask and grown in DMEM media, supplemented with 10% fetal bovine serum (FBS), by incubating the flask at 37 °C in 5% CO_2_. On reaching 80% confluence the cells were harvested by trypsinization and counting was performed in hemocytometer by trypan blue dye exclusion method. The 100 µL of 8 × 10^4^ cells/ mL were seeded in each well of 96-well plate, followed by overnight incubation at 37 °C in a 5% CO_2_. The next day, media was replaced with fresh media containing test compounds with a concentration of 50 µM in 200 µL final volume, and plate was incubated for further 48 hrs. After incubation, the 50 µL of (0.5 mg/mL MTT dye) was added, and plate was incubated for 4 hrs, followed by removal of dye and addition of 100 µL of DMSO in each well to solubilize the formazan crystals. MTT reduction was monitored at 550 nm by using a spectrophotometer. The cell viability was measured to determine the effect of the compounds on cell viability (S1–S4 Files).

### Statistical analysis

SoftMax Pro 4.8 software (Molecular Devices, USA) was used to analyse the data obtained from *in vitro* biochemical assay. Each experiment was performed in triplicate, and results were reported as mean ± standard error mean (SEM). For the determination of IC_50_ values, percent inhibition of enzyme activity (using the formula given earlier) was determined at various concentrations (250–3.125 µM) of the test compounds. This data was plotted as percent inhibition against inhibitor concentration. The IC_50_ values (concentrations of inhibitor required to reduce enzyme activity by 50%) were then calculated by fitting the data to a sigmoidal dose-response curve by Enzyme kinetics software (EZ-FIT), Perrella Scientiﬁc, Inc. (USA). The data acquired from NMR experiments was analyzed through Topspin-Software 4.3.1 (Bruker BioSpin GmbH, Germany).

## Results and discussion

HGXPRT enzyme plays a key role in assisting purine auxotrophs to replicate, and survive. Its inhibition can help to reduce the malarial burden caused by *P. falciparum*. For this purpose, the HGXPRT protein was produced using recombinant technology, as described previously [12,13]. Series of newly synthesized compounds was then screened against the enzyme *via in-vitro* enzyme inhibition assay. Mechanistic and cytotoxic studies were performed *via* computational, biophysical, and MTT assay studies, as explained in detail below;

### Chemistry

Target compounds **1–17** (Fig 1, S1 Table in S2 File) were synthesized by conjugating paroxetine (**a**) with substituted aromatic isothiocyanates (**b**) using Et_3_N as a base *via* straightforward method under reflux conditions with mechanical stirring for 18–20 hours. A silica-based preparative HPLC column (D-60, 20 mm × 250 mm, 5 µm; YMC Co., Ltd., Japan) was used for the purification of compounds with 100% dichloromethane (DCM) as the mobile phase, and detection was performed using UV and refractive index (RI) systems. The same conditions were applied to all compounds to ensure consistency and reproducibility, and similar purification results were obtained. The purity of the isolated compounds was further confirmed by TLC analysis, which showed a single spot for each compound. IR, UV, MS, and NMR techniques were used for structural characterization of all compounds (information provided in supplementary file as Figures M1-143 in S1 File). The ^1^H- and ^13^C-NMR chemical shifts of selected carbothioamide was assigned through DEPT-135°, DEPT-90°, COSY, HSQC, and HMBC spectral analyses. All synthesized compounds **1**–**17** were found to be new. A representative example is presented of compound **3** in Table 1, and Fig 2 for.

**Table 1:** ^1^H-NMR Chemical shift data of representative compound 3.

Positions	*δ* ^1^H (ppm)	Multiplicity (*J* in Hz)	*δ* ^13^C (ppm)
C = S	–	–	180.9
N-H	9.41	s, 1 H, N-H	–
2	5.03, 3.09	d, ^2^*J* = 12.4 Hz, *J*_2/3_ = 11.6 Hz, 2 H, H-2 (a and b)	51.3
3	2.11	br d, 1 H, H-3	41.2
4	3.58	td, *J*_4/5_ = 10.8 Hz, *J*_4/3_ = 4.8 Hz, 1 H, H-4	43.0
5	1.77	(m, 2 H, H-5)	33.2
6	4.84, 3.13	dd, ^2^*J* = 12.0 Hz, *J*_6/5_ = 7.2 Hz, 2 H, H-6 (a, b)	48.7
7	3.58, 3.50	dd, ^2^*J* = 10.0 Hz, *J*_7/3_ = 2.4 Hz, 2 H, H-7	68.7
1´	–	–	141.2
2´	5.88	s, 2 H, H-2	100.9
3´	–	–	147.7
4´	6.44	d, *J*_4´/6´_ = = 2.4 Hz, 1 H, H-4´	97.9
5´	–	–	153.8
6´	4.84	d, ^2^*J* = 12.0 Hz, *J*_2/34_ 11.6 Hz, 2 H, H-2	105.8
7´	6.69	*J*_7´/6´_ = 8.6 Hz, 2 H, H-7´	107.9
1´´	–	–	139.4
2′′, 6′′	7.24	ovp, 2 H, H_-_2′′ and H_-_6′′	129.2
3′′, 5′′	7.06	ovp, 2 H, H_-_3′′ and H_-_5′′	115.2
4′′	–	–	161.8
1′′′	–	–	132.0
2′′′	7.41	s, 1 H, H-2′′′	124.4
3′′′	–	–	142.7
4′′′	7.11	ovp, 1 H, H_-_4′′′	123.6
5′′′	7.30	ovp, 1 H, H_-_5′′′	129.4
6′′′	7.30	ovp, 1 H, H_-_6′′′	123.2

**Fig 1:**
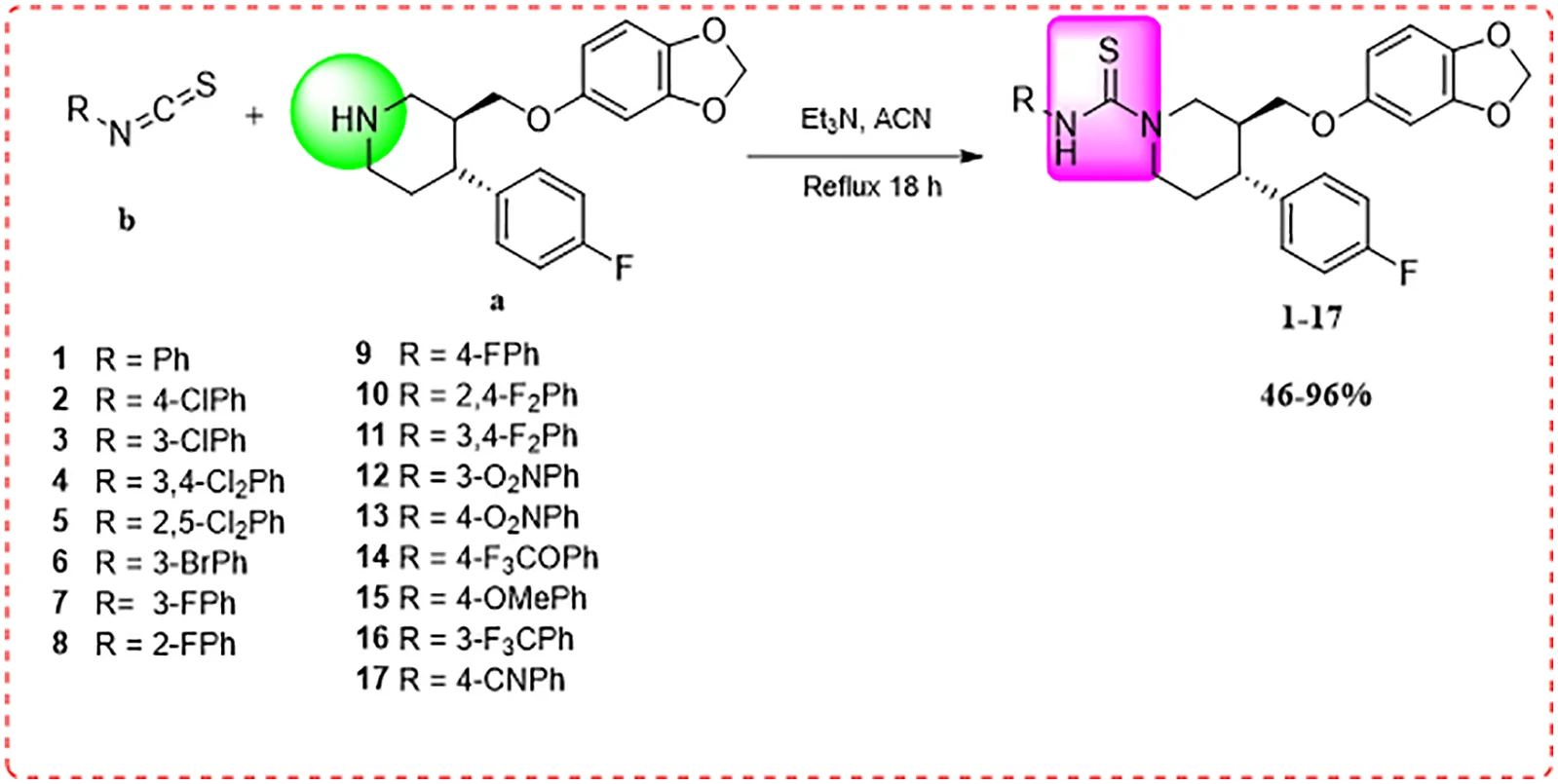
Scheme of synthesis of piperidine-based carbothioamide derivatives 1-17 of paroxetine.

**Fig 2:**
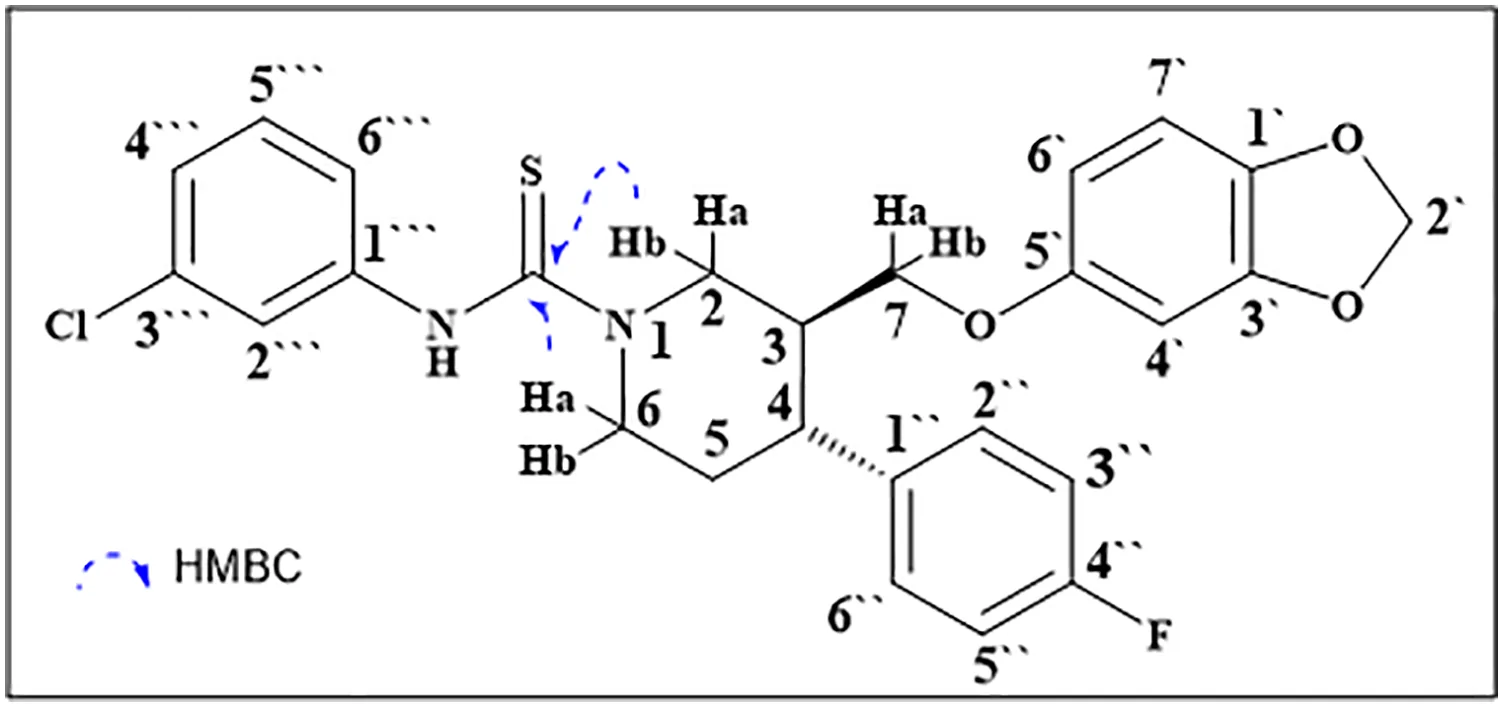
Key HMBC interactions of piperidine-based thiourea analog 3.

The structure of the piperidine based thiourea analog **3** was deduced based on 2D NOESY and HMBC interactions (Fig 1). The HMBC spectrum revealed three bond (J_3_) interactions between the N-H at *δ* 9.41 with C-2′′′, at *δ* 124.4, and C-6′′′ at *δ* 123.2. While the H-6b at *δ* 3.16 and H-2b at *δ* 3.11 showed three bond interactions with quaternary carbon, i.e., C = S at *δ* 180.9. The NOSEY spectrum showed that N-H at *δ* 9.41 interacted with H-2′′′, at *δ* 7.41, H-6′′′, at *δ* 7.30, and H-6a, at *δ* 4.84, which provide detailed structure of analog **3**.

### Enzyme inhibition studies

A total of 17 fully characterized compounds were evaluated against HGXPRT *via* an *in vitro* biochemical inhibition assay. 9-Deazaguanine has been reported to inhibit the activity of HGXPRT with IC_50_ value of 12 ± 1.0 μΜ, hence it was used as a standard [33]. All compounds showed the potential to inhibit HGXPRT enzyme with IC_50_ values between 19 to 76.9 μM, as presented in Fig 3. Among them, compound **13** showed the highest inhibitory activity (IC_50_ = 19 ± 2.09 μM) but was still less potent than the standard (IC_50_ = 12 ± 1.0 μM). Results along with limited structure-activity relationship are presented below:

**Fig 3:**
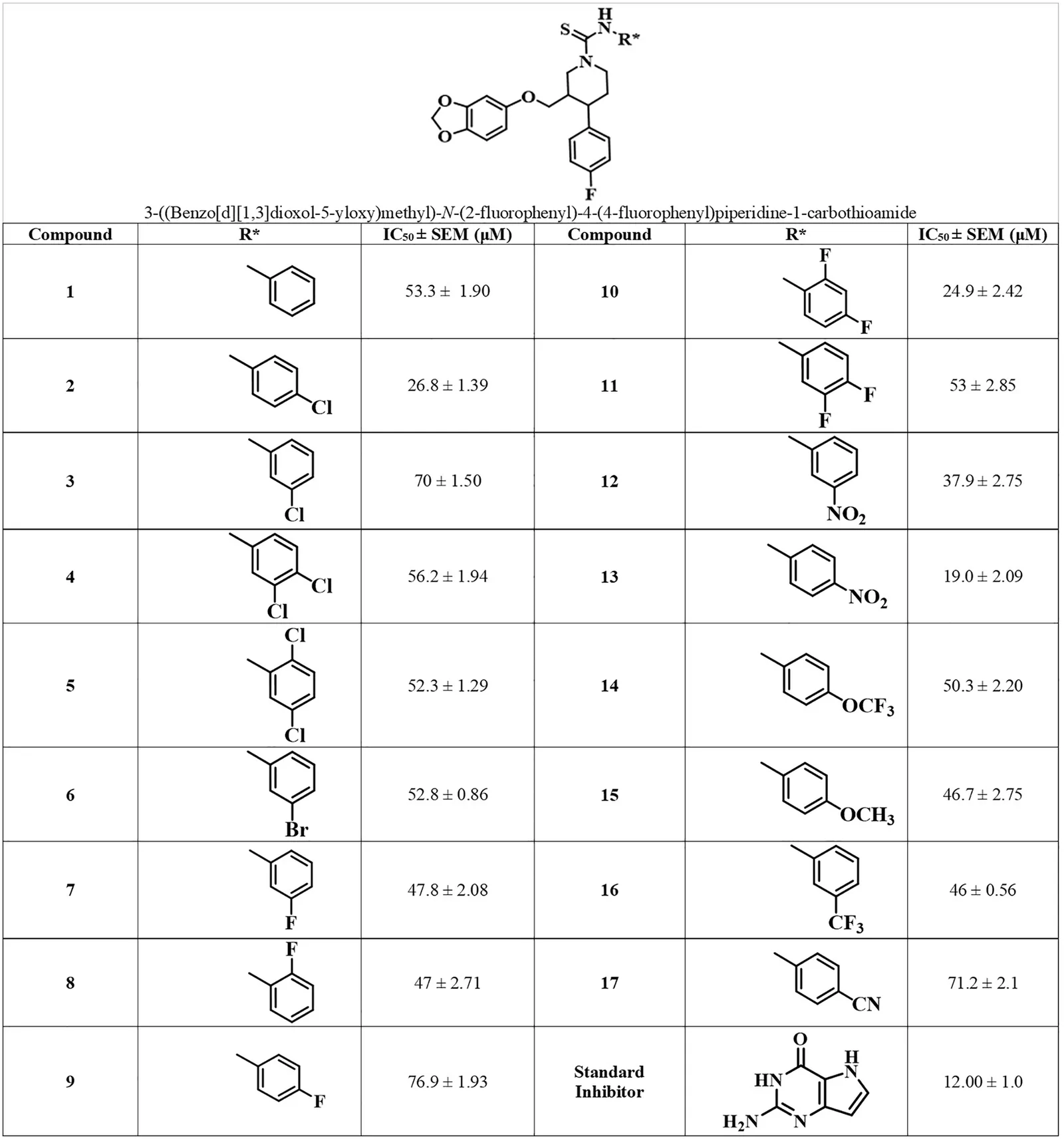
HGXPRT Inhibitory activity of paroxetine-carbothioamide derivatives.

The parent compound **1** of paroxetine-carbothioamide derivatives, with no substitution on the phenyl ring, inhibited 90% activity of the HGXPRT enzyme with IC_50_ of 53.3 ± 1.90 μM. Compounds **2**, and **3** with a mono-chloro group substitution at the *para* and *meta* positions, respectively, showed an increase and decrease in the potency of inhibiting enzyme with IC_50_ values of 26 ± 1.39, and 70 ± 1.50 μM, respectively. Compounds **4**, and **5** with di-chloro substitution also showed an inhibition with IC_50_ values of 56.2 ± 1.94, and 52.3 ± 1.29 μM, respectively. Compound **6** with mono-bromo group substitution at the *meta* position of the phenyl ring showed a good inhibition, with an IC_50_ value 52.8 ± 0.86 μM. The mono-fluoro substituted compounds **7** (*meta*), **8** (*ortho*), and **9** (*para*) showed good to weak HGXPRT inhibition with IC_50_ values of 47.8 ± 2.08, 47 ± 2.71, and 76.9 ± 1.93 μM, respectively. Compounds **10**, and **11** with the di-fluoro group substitution showed IC_50_ values of 24.9 ± 2.42, and 53 ± 2.85 μM, respectively. This indicates that substitution of fluoro group at different positions has a significant impact on the inhibitory activity of test compounds. Compound **12** with substitution of the nitro group at *meta*-position exhibited a good HGXPRT inhibition (IC_50_ = 37.9 ± 2.75 μM). Compound **13** with nitro group at *para*-position exhibited the highest HGXPRT inhibition (IC_50_ = 19 ± 2.09 μM), as compared to parent compound **1** (IC_50_ = 53.3 ± 1.90 μM). Compounds **14**–**17** with substitution of methoxy, trifluoromethoxy, trifluoro, and cyanide groups showed a moderate to weak HGXPRT inhibition with IC_50_ of 50.3 ± 2.20, 46.7 ± 2.75, 46 ± 0.56, and 71.2 ± 2.1 μM, respectively.

### Biophysical studies

Compounds identified as HGXPRT inhibitors in biochemical *in vitro* assay were subjected to STD-NMR studies for the characterization of ligand-protein interactions at atomic level. Based on NOE (Nuclear Overhauser Effect) phenomenon, STD-NMR experiment involves selective saturation of the protein signals in the in the spectral area beneath 0 ppm. This saturation can quickly spread to the entire protein surface *via* spin diffusion, and transferred to the ligand, if it is bound to the protein. When the ligand dissociates from the protein, this saturation is transferred into the solution [17].

Data embedded in the STD-NMR experiment can be used to map the orientation of the ligand on protein surface, *i.e.,* group epitope mapping (GEM). In GEM analysis, proton(s) with highest STD integral value is randomly assigned a value of 100%, and STD integrals of all other protons are adjusted in relation to it. This provides the relative levels of saturation received by other protons. The degree of saturation indicates the proximity of ligand protons to the protein surface, with ligand protons in close proximity to protein exhibiting a greater saturation than those further away (> 5 Å) [17].

For the STD-NMR experiments, compounds **2**, **4**, and **6** were selected based on their enzyme inhibitory activity (ranging from strong to weak) and adequate solubility in aqueous buffer. The H-4’ of benzodioxol moiety of compound **2** showed the highest STD integral, therefore it was assigned to receive the maximum saturation (100%) from the protein. The H-2’/H-6’/H-7’ showed the 92−98% relative saturation, indicating the close proximity of benzodioxol ring of compound **2** to the protein. This was followed by the H-2”/H-6” and H-3”/ H-5” of fluorophenyl group of compound **2** that displayed 69% to 77% of relative saturation. While the protons of the phenyl substituted on carbothioamide moiety conferred the 59% relative saturation. GEM analysis proposed that compound **2** is interacting with protein, mainly *via* benzodioxol group of paroxetine (Fig 4).

**Fig 4:**
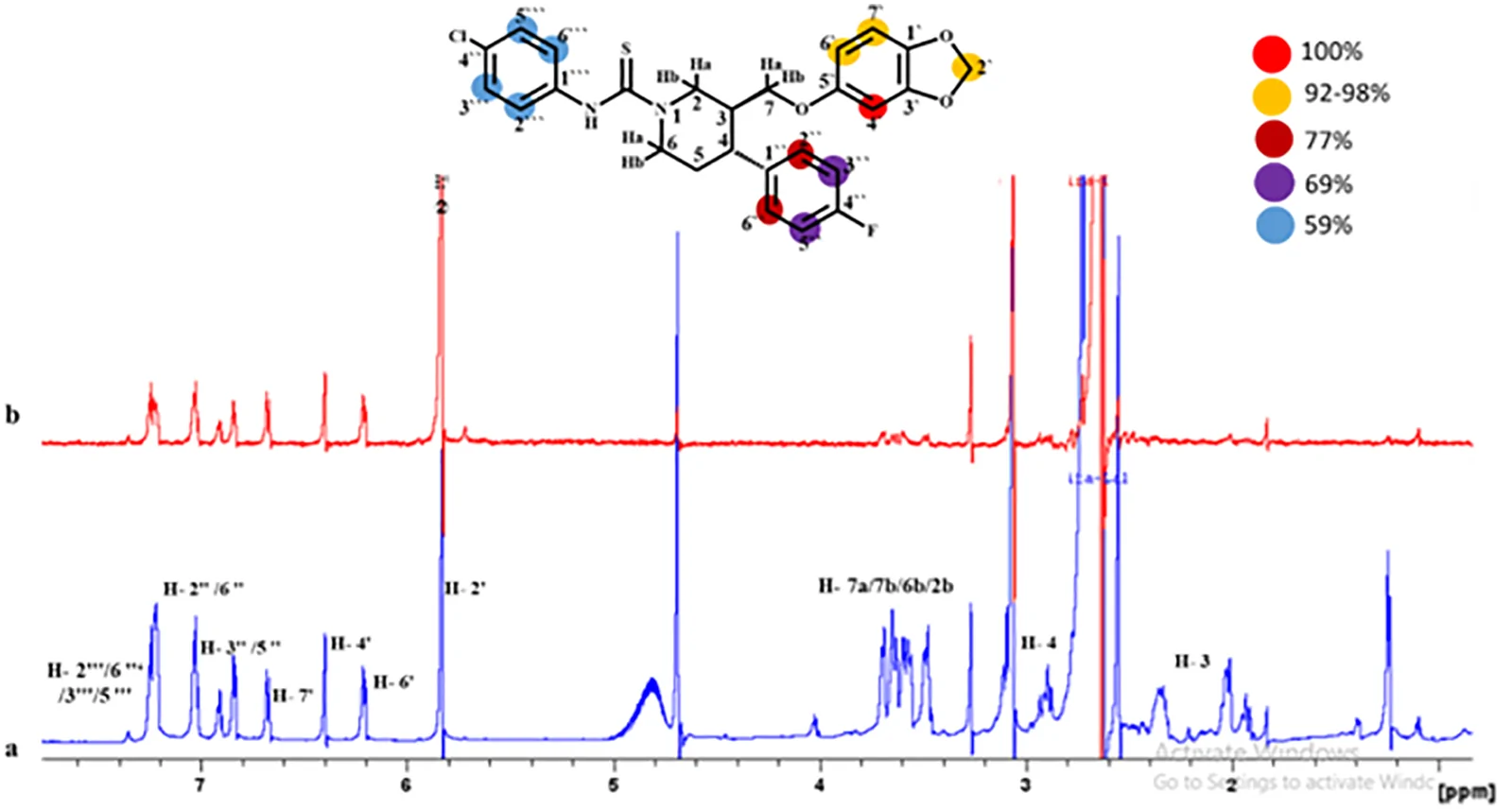
STD-NMR Analysis of compound 2. a) ^1^H-NMR (off-resonance) spectrum (blue) of compound 2. b) STD difference spectrum in the presence of HGXPRT protein (red); Relative saturation (%) of signals normalized to that of the H4’ signal is indicated with a color code.

STD-NMR analysis carried out for the compounds **4**, and **6** showed that the benzodioxol and fluorophenyl groups of paroxetine and phenyl substituted carbothioamide moiety also showed interactions with the protein, as can be seen in the difference spectra (S1 and S2 Figs in S2 File).

### Molecular docking studies

All the active compounds were finally subjected to the molecular docking studies to gain insights into ligand-receptor interactions. HGXPRT enzyme is a homo-tetrameric protein with catalytic site in each subunit. The catalytic site contained binding sites for purine base, ribose moiety, phosphate, and pyrophosphate/magnesium. In our previous studies, we validated the catalytic site by redocking the co-crystallized ligand, achieving a docking score of −6.922 [13]. The new series of compounds were unable to fit in the same catalytic-centred-grid, likely due to steric hindrance, scaffold geometry, and altered charge distribution. Therefore, SiteMap analysis was used to identify five potential binding sites across two homologous protein chains. Sites 1 and 2, as well as 4 and 5, are homologous pairs, while site 3 is at the interface of the chains. Despite high scores, sites 1 and 2 were distant from the catalytic domain; hence, site 4 near the pyrophosphate site was chosen for receptor grid generation (S2 Table in S2 File). All the compounds were able to occupy this site, except compound **10** that showed an unfavorable docking score (0). Hence, this compound was redocked at site 3 (Fig 5A-E). The docking scores, and binding energies are presented in Fig 6, and S3 Table in S2 File.

**Fig 5:**
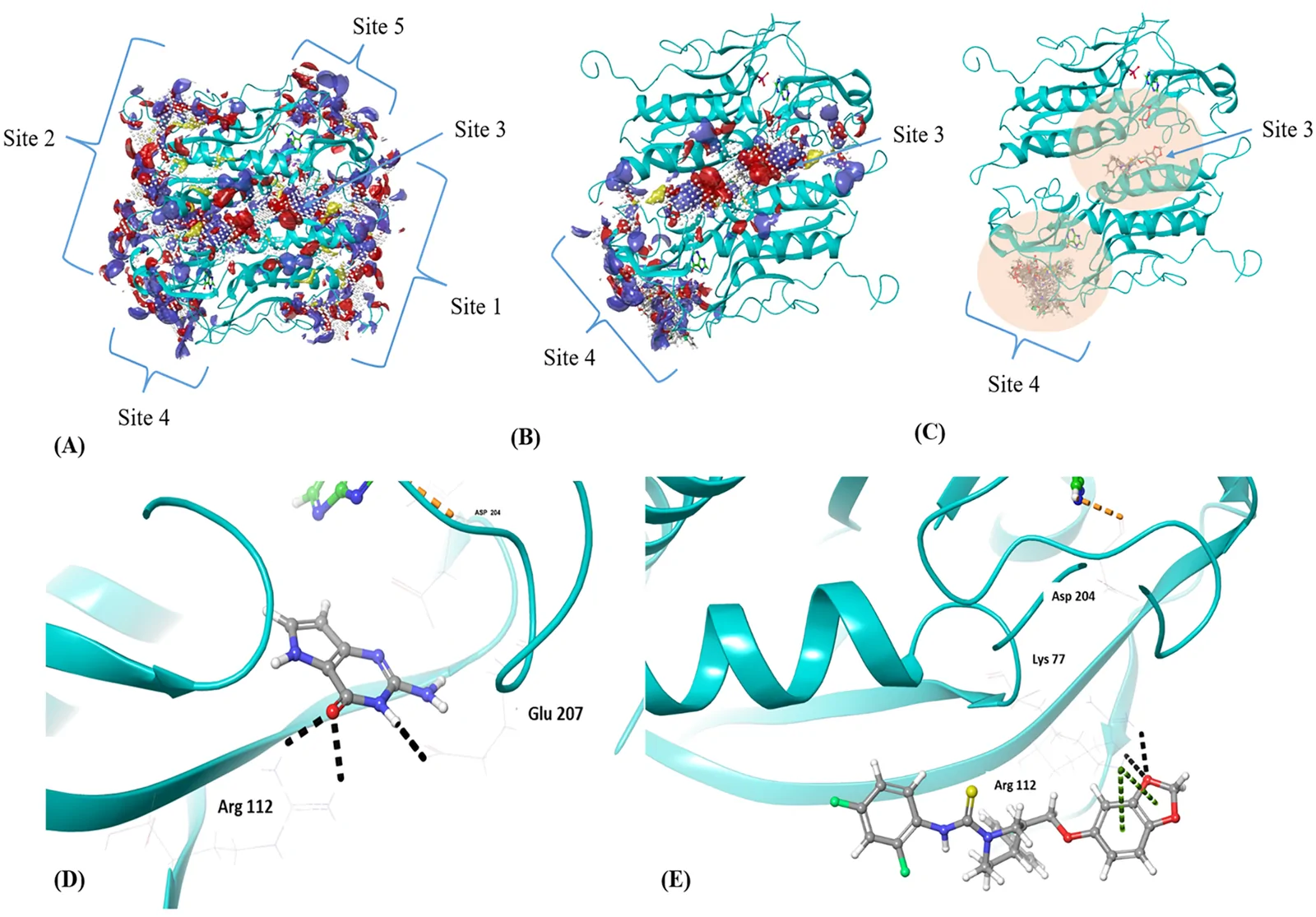
(A) SiteMap analysis of 3OZF protein. (B) Site 4 and 3 closes to Pyrophosphate site. (C) Docked pose view of **1**-**17** compounds at site 4. Site 3 indicating re-docking of compound **10**. (D) Ligand-residue interaction diagram of 3OZF protein with standard ligand 9-deazaguanine, docked in pyrophosphate grid. (E) Ligand-residue interaction diagram of compound **10**, docked in a Site 3.

**Fig 6:**
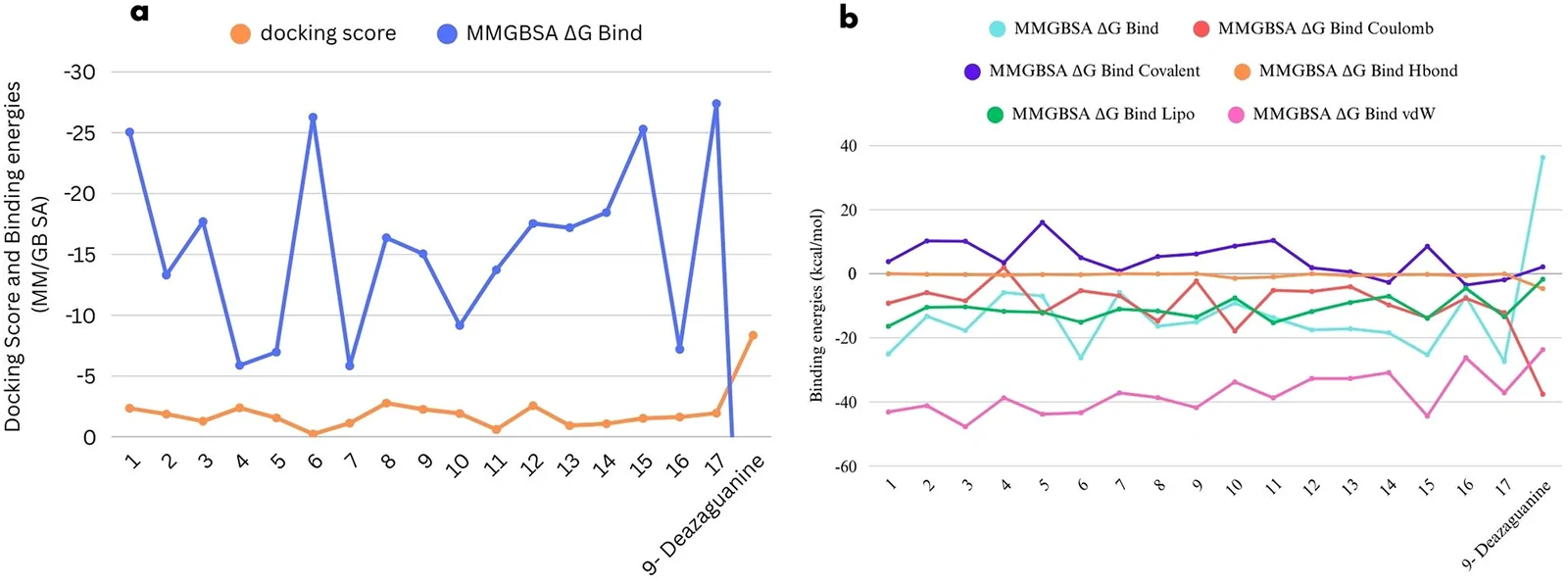
(a) The line graph illustrates molecular docking scores and MM/GBSA ΔG binding energies for compounds 1–17 complexed with HGXPRT enzyme, and (b) highlighting the roles of columbic, covalent, hydrogen bond, hydrophobic, and van der Waals interactions.

Compound **1** showed π-π stacking with His71, and aromatic hydrogen bonding with Asp206 (Fig 7a). All the compounds **2**–**5** with mono-, and di-chloro substitutions showed a π- cationic interaction with Lys103. In addition to this, compound **2** showed an aromatic hydrogen bond with Tyr226, and π-π stacking interaction with His71 (Fig 7b). Whereas compound **3** was able to form four aromatic hydrogen bonds with Lys227, Glu269, and Tyr39 of enzyme (Fig 7c).

**Fig 7:**
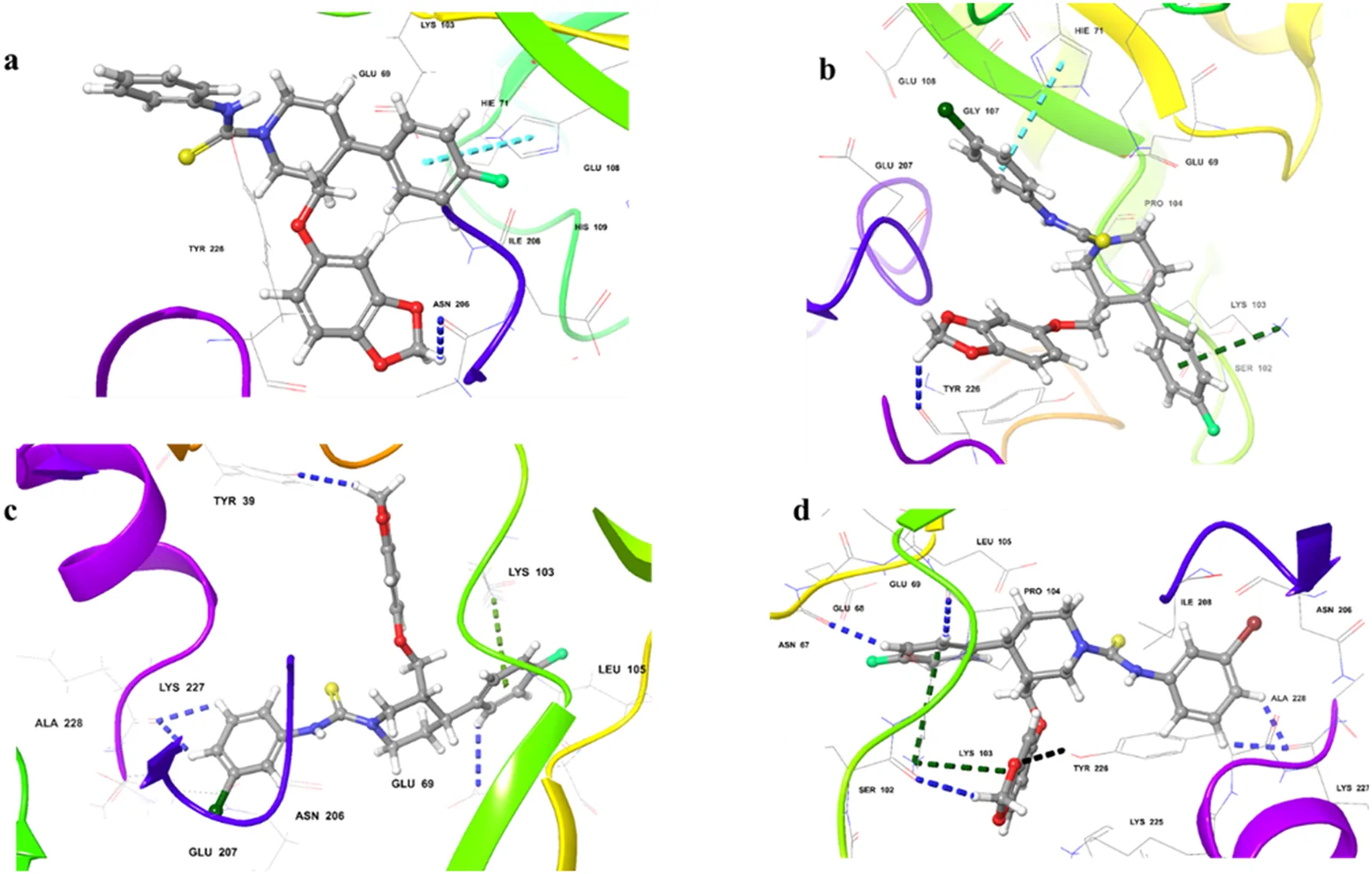
3D Docked poses of compounds (a) 1 (b) 2, (c) 3, and (d) 4 in the allosteric site of HGXPRT. Black color represents hydrogen bond, blue color indicates aromatic hydrogen bond, while cyan color indicates π-π stacking interactions, while green color indicates π-cationic interactions.

Compounds **4**, and **5** showed similar non-covalent interactions, including π-π stacking, and π-cationic interactions with His71, and Lys225, respectively. Moreover, compound **4** showed hydrogen and aromatic hydrogen bondings with Glu108, and Lys225, respectively (Fig 7d). While compound **5** showed hydrogen and aromatic hydrogen bonds with Glu69, and Glu68, respectively. The chloro group at *meta* position was able to form a halogen bond with Glu108 (S3a Fig in S2 File).

Compound **6** with Br at *meta* positions showed five aromatic hydrogen bonds with Lys227, Asp67, Pro104, and Ser102. Along with this a hydrogen bond with Tyr226 was also observed. The compound also forms a π-cationic interaction with Lys103 (S3b Fig in S2 File). Compound **7** formed three aromatic hydrogen bonds with Asp67, Glu69, and Pro104. Along with this, it also formed a π-cationic interaction with Lys103, and Lys138 (S3c Fig in S2 File). Compound **8** formed four aromatic hydrogen bonds with Asp67, Asp206, Pro104, and Lys227, as well as a hydrogen bond with Lys138. It also interacted *via* π-π stacking, and π-cationic interactions with His71, and Lys103, respectively (S3d Fig in S2 File). Compound **9** with *para* fluoro substituted group interacted with the enzyme *via* hydrophobic interactions with Ile 208, Tyr 226, Tyr 39, Lys 225, and Lys 103 (S4a Fig in S2 File). As compound 10 showed a docking score of 0 at site 4. Consequently, it was re-docked to a newly created grid close to the pyrophosphate site, identified as site 3, yielding a docking score of −2.094 with respect to standard which showed a score of −4.758 and forming noncovalent interactions with Glu 207 and Arg 112 (S10C-E Fig in S2 File). Compound **11** showed four π-cationic interactions with Lys138, Lys225, and Lys103 (S4c Fig in S2 File).

Compounds **12**, and **13** with nitro substitutions showed multiple non-covalent interactions, including four aromatic hydrogen bonds with Asp206, Glu207, Asp67, Glu68, π-cationic interactions with Lys103, π-π stacking with His71, and two salt bridges with Lys103, and Glu68 (S4d Fig in S2 File). Similarly, compound **13** showed two salt bridges with Glu108, and Glyu207, along with hydrogen bonding with Glu69, and Glu108. It also interacted with His71 *via* π-π stacking (S5a Fig in S2 File). Compound **14** formed three aromatic hydrogen bonds with Tyr226, Pro104, and Asp69, and a π-cationic interaction with Lys103 (S5b Fig in S2 File). While, compound **15** showed two aromatic hydrogen bonds with Pro104, and two π-cationic interactions with Lys103, and Lys138 (S5c Fig in S2 File). Compound **16** showed hydrogen and aromatic hydrogen bonds with Tyr226, Ser102, and Lys225, respectively (S5d Fig in S2 File). A π-cationic interaction was observed with Lys225. Compound **17** showed two aromatic hydrogen bonds with Cys134, and His71, and a π-cationic interaction with Lys138 (S5e Fig in S2 File).

The docking studies predicted the binding of all the newly identified HGXPRT inhibitors in the allosteric site. They mainly interacted *via* π-π stacking, and π-cationic interactions with His71 and Lys103, respectively. These newly identified hits require further *via in-vitro,* and pre-clinical studies.

### ADMET analysis

ADMET profiling was run using Maestro’s QikProp tool, and 11 distinct pharmacokinetic and toxicity-related parameters were reported (S4 Table in S2 File). The evaluated parameters include Lipinski’s rule of 5 (molecular weight, donor/acceptor hydrogen bonds, rule of 5), water solubility (QPLogpo/w), intestinal absorption (Caco-2 cell permeability), skin permeability (Log kp, cm/s), plasma protein binding (QPlogKhsa), crossing the blood-brain barrier (QPLogBB), cardiac toxicity (QPLogHERG), and kidney epithelial cells (QPPMDCK). Each parameter was evaluated based on acceptable threshold values (S4 Table in S2 File). Each parameter was assigned a binary score, where a value of 1 indicated compliance with well-known suitable thresholds, and a value of 0 denoted deviation from these criteria (Fig 8). The total ADMET percentage was then determined by using a simple percentage formula:

**Fig 8:**
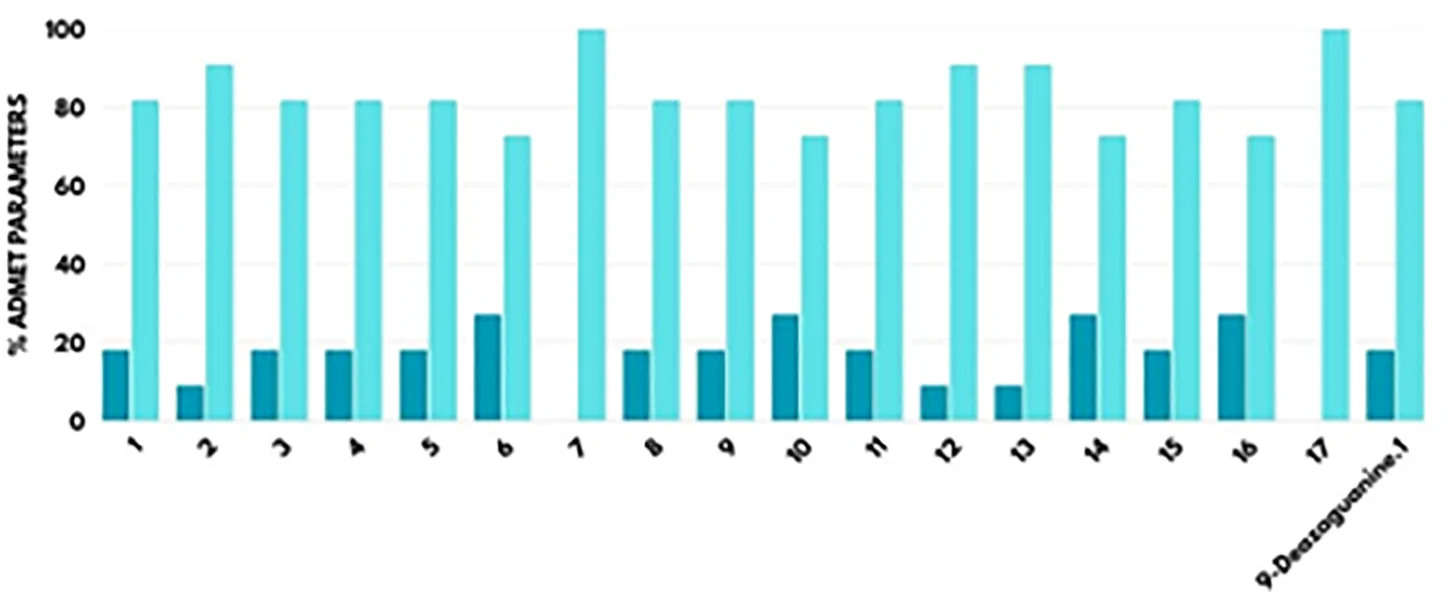
Percentage ADMET profiling of all compounds, and the standard inhibitor, 9-deazaguanine. Dark blue bars represent the percentage of violated ADMET parameters, while light blue bars indicate the parameters evaluated. The figure also reflects the overall safety profile in percentage terms.


$$\begin{array}{c} \%ADMETProfile=\frac{\#ofparametersoutofacceptablerange}{Total\#ofevaluatedparameters(n=\text{1}\text{1})}\times \text{1}\text{0}\text{0} \end{array}$$

In general, the compounds display average pharmacokinetic characteristics with significant drawbacks (S4 Table in S2 File). A few compounds (**7**, **11**, **12**, **13**, and **17**) indicate anticipated solubility problems, and 9 of the 17 compounds do not meet Lipinski’s rules. These results reveal trade-offs in physicochemical characteristics, especially between lipophilicity and solubility, indicating that additional structural optimization is necessary during further studies. (Fig 7, Table S3 in S2 File). The analysis showed that all the compounds, exceeded the QPlogPo/w threshold. This parameter assesses lipophilicity by calculating the logarithmic partition coefficient between octanol and water to predict solubility. The standard (9-deazaguanine) was below the threshold, while the remaining compounds were above the reference range (–2.0 to 6.5) (S5 Table in S2 File).

### Molecular dynamic (MD) simulations

To assess the conformational stability of hypoxanthine-guanine-xanthine phosphoribosyl transferase (HGXPRT) with its identified inhibitors, MD simulations were performed for 200 ns. Ligand stability parameters are presented in S6 Fig in S2 File. The protein’s Cα backbone, and ligand RMSD were calculated. The standard inhibitor 9-deazaguanine showed stable structural conformation throughout 200 ns of trajectory time with a RMSD value <3 A°. Likewise, protein’s Cα backbone also remained structurally stable throughout simulation within acceptable range 1.3 A°. Residue Val198 maintained hydrogen interaction for longest duration of simulation time, i.e., 200 ns (S6 Fig in S2 File), followed by Asp204 which continued cumulative interaction fraction of >1.5 of hydrogen and water bridges (S7 Fig in S2 File).

The molecular dynamics studies of compound **2** also indicated a stable protein-ligand conformation throughout simulation trajectory with a 2.2 Å protein’s RMSD value, followed by 0.3 Å RMSD value of ligand over the trajectory time. This indicated that enzyme-inhibitor complex remained stable under virtual physiological parameters, without significant displacement (S8 Fig in S2 File). The system achieved dynamic equilibrium without large structural perturbations. Similarly, compound **12** showed protein-ligand (PL) RMSD within acceptable range (<2 A°). The PL-RMSD profile collectively confirms the arrangement of a stable and well-balanced enzyme-inhibitor complex under physiological simulation settings. The residue interaction histogram showed the persistency of catalytically important residues (S9 Fig in S2 File). In compound **2,** notably residues such as Pro104, Lys138, and Ile208 maintained hydrophobic, hydrogen and water-mediated hydrogen bonds emphasizing their critical role in ligand equilibrium (S8 Fig in S2 File). Similarly, in compound **12**, His71 formed hydrophobic interaction for >80% of simulation time (S8 Fig in S2 File).

As the docking score was low at site 4 for compound **10**. The glide grid was generated again on a different site, i.e., site 3 (Fig 5C). the analyses of enzyme-inhibitor interactions showed that the protein RMSD value consistently remained within range of 1.5 Å, indicating atomic movements are within an acceptable limit. Followed by ligand RMSD (S10 Fig in S2 File) of 4.5 Å. Tyr116 exhibited a stable hydrophobic interaction with an interaction fraction of 0.8 (160 ns), followed by Asn206, which formed hydrogen and water bridges for about 120 ns, then Tyr205 for 80 ns during the simulation period, indicating sustained enzyme-inhibitor conformation over the course of the simulation time.

### Cytotoxicity studies

All the active compounds were found non-cytotoxic at the tested concentration toward human fibroblast (BJ) cells, maintaining > 86% cell viability of BJ cells, except compound **6** which showed 74% cell viability as presented in S5 Table in S2 File and Fig 9).

**Fig 9:**
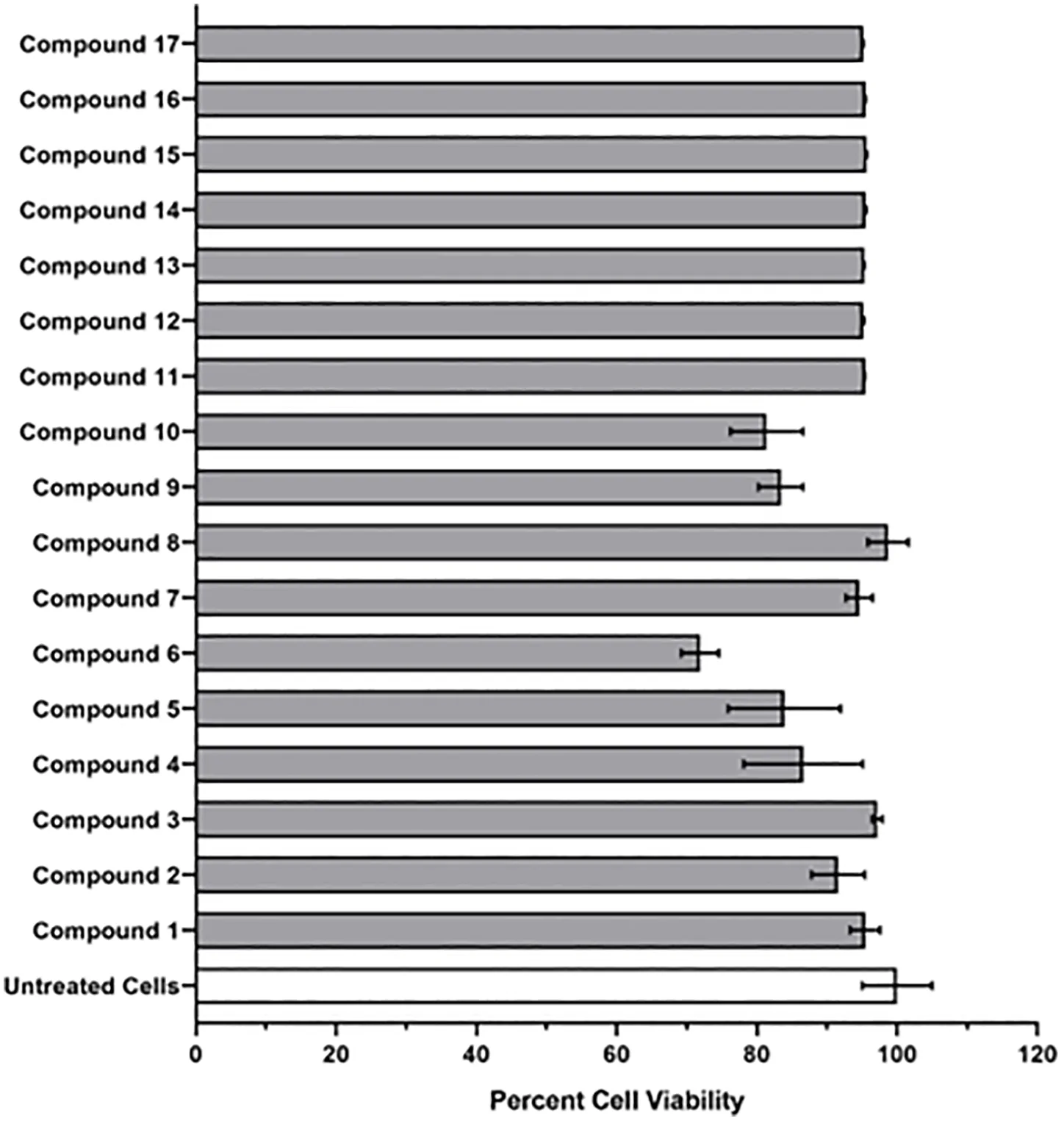
Percent cell viability in the presence of active compounds *via* MTT assay. Statistical analysis was performed by student t test, and no significant differences were observed among the tested compounds and untreated cells (p > 0.05).

## Conclusion

Inhibition of hypoxanthine-guanine-xanthine phosphoribosyltransferase (HGXPRT) enzyme is an important approach for drug discovery against infections caused by purine auxotrophs. These include malaria caused by *P. falciparum*. The HGXPRT is relatively a new target, potentially less prone to existing resistance mechanisms observed against other anti-parasitic drugs. The present study was designed to identify the HGXPRT inhibitors, and for this purpose 17 paroxetine-carbothioamide derivatives were synthesized, and evaluated. Interestingly all these compounds showed a good to weak enzyme inhibitory potential. Bio-physical, and computational studies were further carried out to get insights into enzyme-ligand interactions, and complex stability. Non-cytotoxic HGXPRT inhibitors identified from the current study, can be further studied for anti-parasitic drug discovery. These potential inhibitors could also be used as adjuvants, combined with existing antimalarial drugs for enhanced efficacy.

## Supporting information

S1 File Spectroscopic characterization (IR, UV, NMR, MS) of synthesized compounds 1–17. (DOCX)

S2 File STD-NMR, molecular docking, and simulation data of compounds. (DOCX)

S3 File HPLC Chromatograms of compounds. (PDF)

S4 File Spectrophotometer raw data for enzyme assay. (XLSX)
