# Supplementary material for: Carbothioamide functionalized paroxetine analogues as antiparasitic agents targeting the hypoxanthine-guanine-xanthine phosphoribosyltransferase enzyme: Synthesis, characterization, biochemical, biophysical, and in silico studies
Source: PLoS One. 2026 Oct 22;21(10):e0359519. doi: 10.1371/journal.pone.0359519 (PMC13690214; doi:10.1371/journal.pone.0359519)
Supplement: Supplementary file 1 [file pone.0359519.s003.pdf]

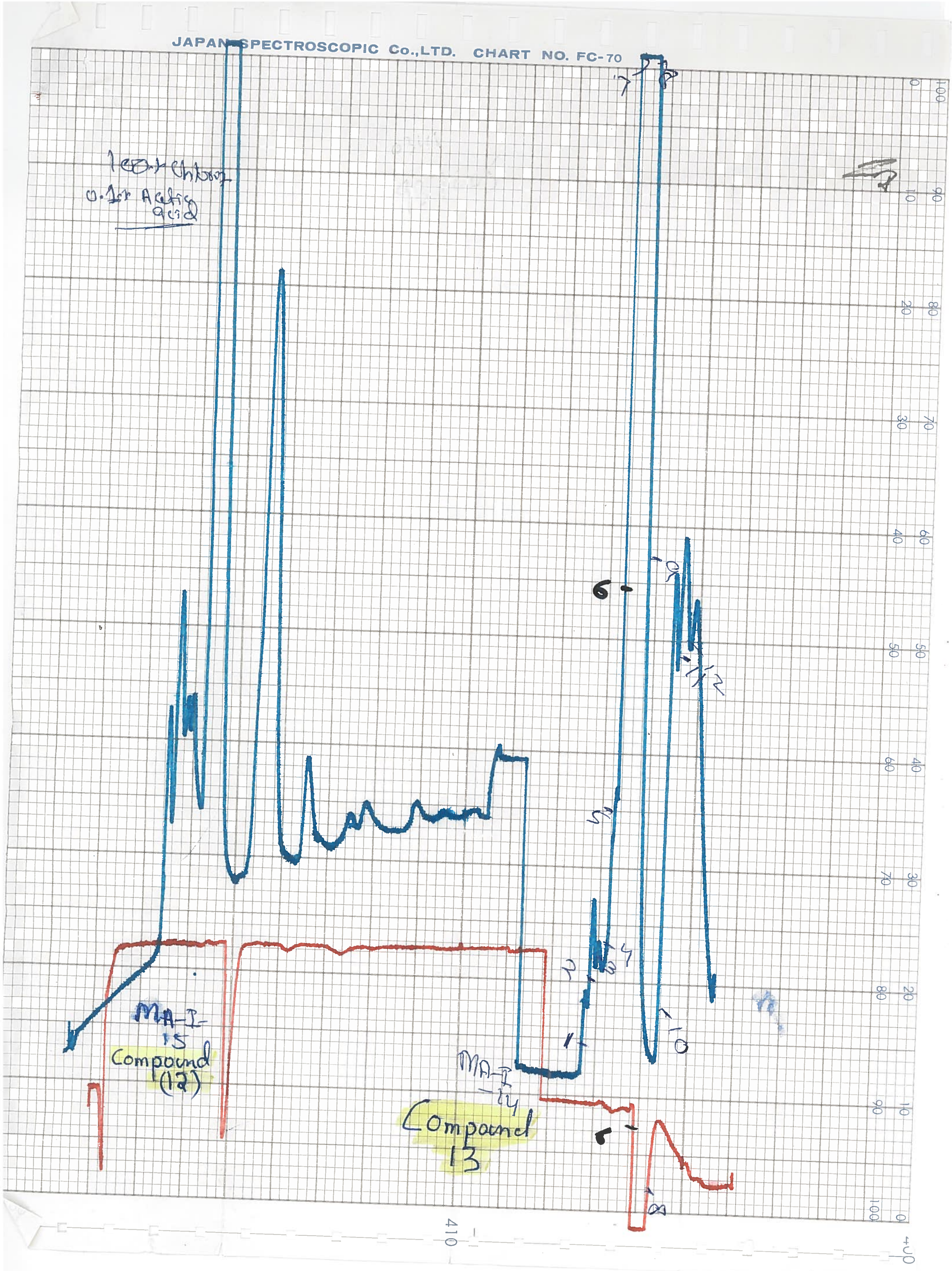
JAPAN SPECTROSCOPIC Co.,LTD. CHART NO. FC-70
MA-I-15
Compound (12)
MA-I-14
Compound 13
410

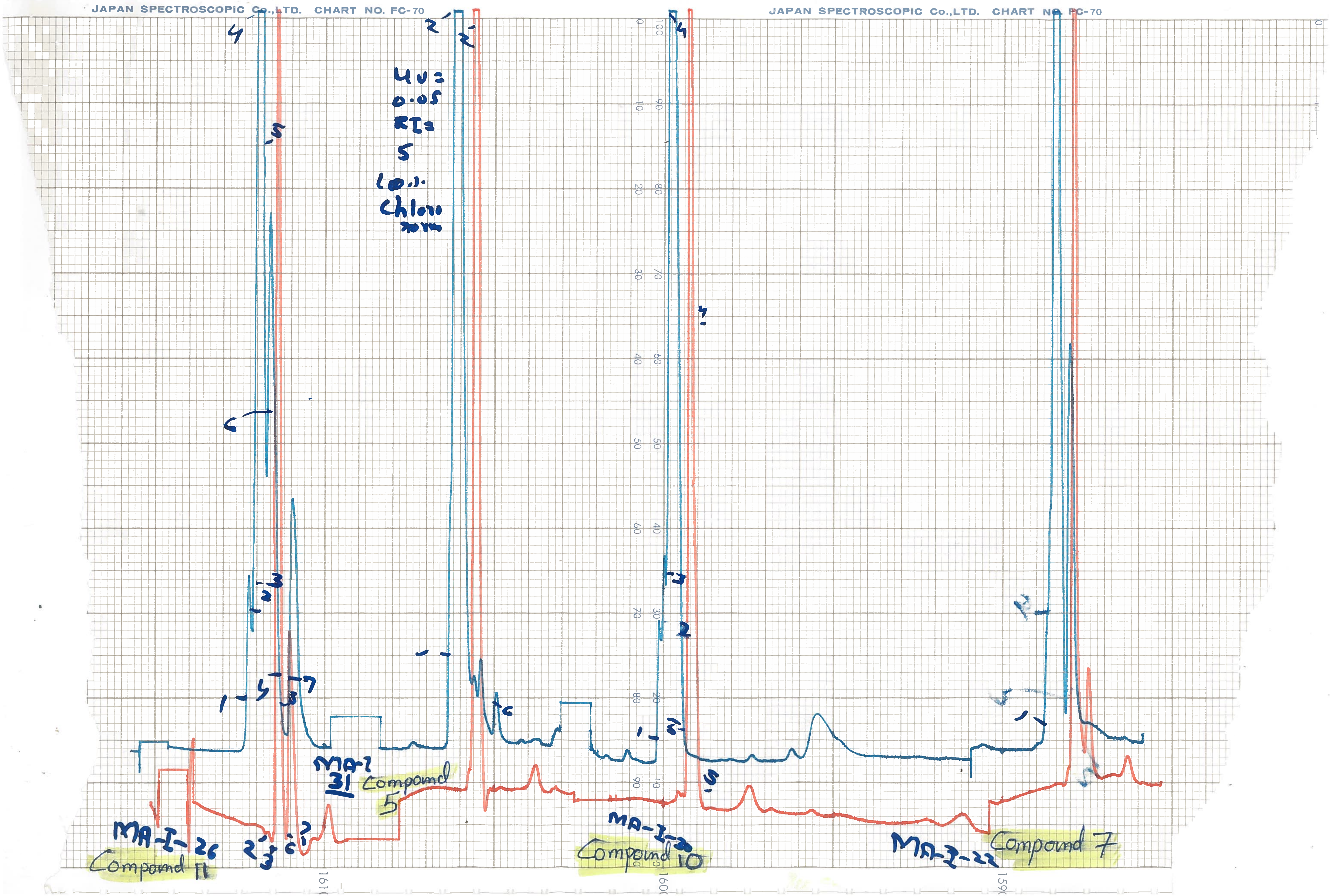
JAPAN SPECTROSCOPIC Co.,LTD. CHART NO. FC-70
JAPAN SPECTROSCOPIC Co.,LTD. CHART NO. FC-70
UV= 0.05
RI= 5
Chloroform
MA-I 31 Compound 5
MA-I-26 Compound 11
MA-I-20 Compound 10
MA-I-22 Compound 7

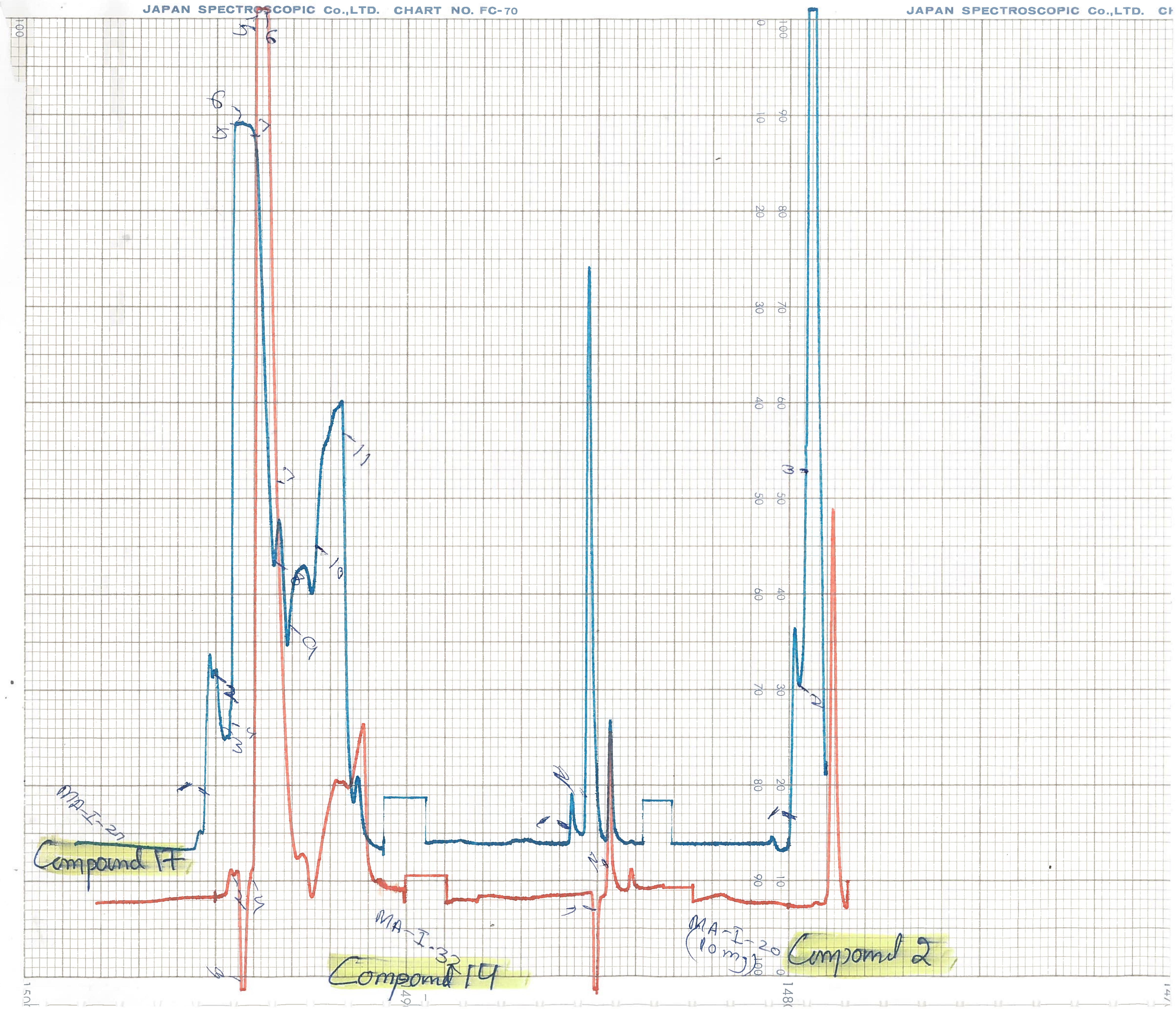
JAPAN SPECTROSCOPIC Co.,LTD. CHART NO. FC-70
JAPAN SPECTROSCOPIC Co.,LTD.
MA-I-27
Compound 17
MA-I-32
Compound 14
MA-I-20
(10 mg)
Compound 2

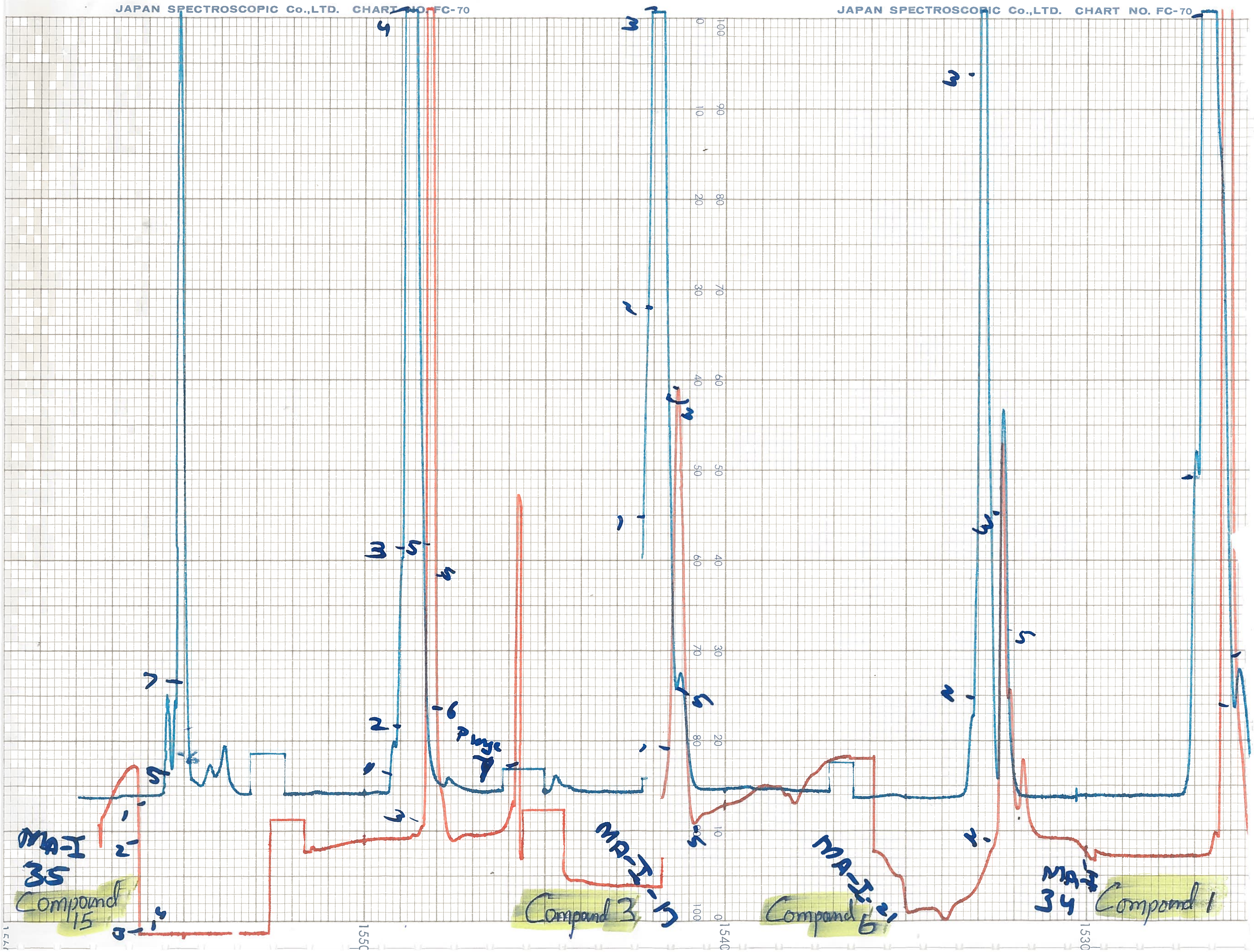
JAPAN SPECTROSCOPIC Co.,LTD. CHART NO. FC-70
JAPAN SPECTROSCOPIC Co.,LTD. CHART NO. FC-70
MA-I 35
Compound 15
purge
Compound 3
Compound 6
MA-I 34
Compound 1
1550
1540
1530
