# Supplementary material for: Carbothioamide functionalized paroxetine analogues as antiparasitic agents targeting the hypoxanthine-guanine-xanthine phosphoribosyltransferase enzyme: Synthesis, characterization, biochemical, biophysical, and in silico studies
Source: PLoS One. 2026 Oct 22;21(10):e0359519. doi: 10.1371/journal.pone.0359519 (PMC13690214; doi:10.1371/journal.pone.0359519)
Supplement: Supplementary file 2 [file pone.0359519.s001.docx]

Supplementary File 1

**Title: Carbothioamide Functionalized Paroxetine Analogues as Antiparasitic Agents Targeting the Hypoxanthine-Guanine-Xanthine Phosphoribosyltransferase Enzyme: Synthesis, Characterization, Biochemical, Biophysical, and *in Silico* Studies**

| **Sr. No.** | **Contents** | **Pag#** |
| --- | --- | --- |
| **Figure M1**  | IR spectrum of compound **1** | 11 |
| **Figure M2**  | Uv spectrum of compound **1** | 12 |
| **Figure M3**  | ^1^H NMR spectrum of compound **1** | 13 |
| **Figure M4**  | Expansion of part (δ 9.5 – 5.9) of ^1^H NMR spectrum of compound **1** | 14 |
| **Figure M5**  | Expansion of part (δ 5.0 – 1.7) of ^1^H NMR spectrum of compound **1** | 15 |
| **Figure M6**  | Expansion of part (δ 3.6 – 1.7) of ^1^H NMR spectrum of compound **1** | 16 |
| **Figure M7**  | ESIMS of compound **1** | 17 |
| **Figure M8**  | HR-FAB-MS of compound **1** | 18 |
| **Figure M9**  | IR spectrum of compound **2** | 19 |
| **Figure M10**  | Uv spectrum of compound **2** | 20 |
| **Figure M11**  | ^1^H NMR spectrum of compound **2** | 21 |
| **Figure M12**  | Expansion of part (δ 9.5 – 5.9) of ^1^H NMR spectrum of compound **2** | 22 |
| **Figure M13**  | Expansion of part (δ 5.0 – 1.7) of ^1^H NMR spectrum of compound **2** | 23 |
| **Figure M14**  | EIMS of compound **2** | 24 |

| **Figure M15**  | HR-FAB-MS of compound **2** | 25 |
| --- | --- | --- |
| **Figure M16**  | IR spectrum of compound **3** | 26 |
| **Figure M17**  | Uv spectrum of compound **3** | 27 |
| **Figure M18**  | ^1^H NMR spectrum of compound **3** | 28 |
| **Figure M19**  | Expansion of part (δ 7.4 – 4.8) of ^1^H NMR spectrum of compound **3** | 29 |
| **Figure M20**  | Expansion of part (δ 3.6 – 1.7) of ^1^H NMR spectrum of compound **3** | 30 |
| **Figure M21**  | ^13^C NMR spectrum of compound **3** | 31 |
| **Figure M22**  | Expansion of part (δ 161 – 129) of ^13^C NMR spectrum of compound **3** | 32 |
| **Figure M23**  | Expansion of part (δ 124 – 41) of ^13^C NMR spectrum of compound **3** | 33 |
| **Figure M24**  | Expansion of part (δ 41 – 33) of ^13^C NMR spectrum of compound **3** | 34 |
| **Figure M25**  | DEPT-90 NMR spectrum of compound **3** | 35 |
| **Figure M26**  | Expansion of part (δ 129 – 97) of DEPT-90 NMR spectrum of compound  **3** | 36 |
| **Figure M27**  | DEPT-135 NMR spectrum of compound **3** | 37 |
| **Figure M28**  | Expansion of part (δ 130 – 100) of DEPT-135 NMR spectrum of compound **3** | 38 |
| **Figure M29**  | Expansion of part (δ 66 – 33) of DEPT-135 NMR spectrum of compound  **3** | 39 |

| **Figure M30**  | COSY NMR spectrum of compound **3** | 40 |
| --- | --- | --- |
| **Figure M31**  | HMBC NMR spectrum of compound **3** | 41 |
| **Figure M32**  | Expansion of part (δ 9.5 – 2.0) of HMBC NMR spectrum of compound **3** | 42 |
| **Figure M33**  | Expansion of part (δ 7.6 – 5.7) of HMBC NMR spectrum of compound **3** | 43 |
| **Figure M34**  | HSQC NMR spectrum of compound **3** | 44 |
| **Figure M35**  | NOESY NMR spectrum of compound **3** | 45 |
| **Figure M36**  | Expansion NOESY NMR spectrum of compound **3** | 46 |
| **Figure M37**  | ESIMS of compound **3** | 47 |
| **Figure M38**  | Expansion of ESIMS of compound **3** | 48 |
| **Figure M39**  | HR-FAB-MS of compound **3** | 49 |
| **Figure M40**  | IR spectrum of compound **4** | 50 |
| **Figure M41**  | Uv spectrum of compound **4** | 51 |
| **Figure M42**  | ^1^H NMR spectrum of compound **4** | 52 |
| **Figure M43**  | Expansion of part (δ 7.6 – 4.8) of ^1^H NMR spectrum of compound **4** | 53 |
| **Figure M44**  | Expansion of part (δ 3.9 – 1.8) of ^1^H NMR spectrum of compound **4** | 54 |
| **Figure M45**  | ESIMS of compound **4** | 55 |
| **Figure M46**  | Expansion of ESIMS spectrum of compound **4** | 56 |

| **Figure M47**  | HR-FAB-MS of compound **4** | 57 |
| --- | --- | --- |
| **Figure M48**  | IR spectrum of compound **5** | 58 |
| **Figure M49**  | Uv spectrum of compound **5** | 59 |
| **Figure M50**  | ^1^H NMR spectrum of compound **5** | 60 |
| **Figure M51**  | Expansion of part (δ 7.5 – 4.8) of ^1^H NMR spectrum of compound **5** | 61 |
| **Figure M52**  | Expansion of part (δ 3.6 – 1.7) of ^1^H NMR spectrum of compound **5** | 62 |
| **Figure M53**  | ESIMS of compound **5** | 63 |
| **Figure M54**  | Expansion of ESIMS spectrum of compound **5** | 64 |
| **Figure M55**  | HR-FAB-MS of compound **5** | 65 |
| **Figure M56**  | IR spectrum of compound **6** | 66 |
| **Figure M57**  | Uv spectrum of compound **6** | 67 |
| **Figure M58**  | ^1^H NMR spectrum of compound **6** | 68 |
| **Figure M59**  | Expansion of part (δ 9.5 – 5.9) of ^1^H NMR spectrum of compound **6** | 69 |
| **Figure M60**  | Expansion of part (δ 5.0 – 1.8) of ^1^H NMR spectrum of compound **6** | 70 |
| **Figure M61**  | ESIMS of compound **6** | 71 |
| **Figure M62**  | HR-FAB-MS of compound **6** | 72 |
| **Figure M63**  | IR spectrum of compound **7** | 73 |

| **Figure M64**  | Uv spectrum of compound **7** | 74 |
| --- | --- | --- |
| **Figure M65**  | ^1^H NMR spectrum of compound **7** | 75 |
| **Figure M66**  | Expansion of part (δ 7.3 – 5.9) of ^1^H NMR spectrum of compound **7** | 76 |
| **Figure M67**  | ESIMS of compound **7** | 77 |
| **Figure M68**  | HR-FAB-MS of compound **7** | 78 |
| **Figure M69**  | IR spectrum of compound **8** | 79 |
| **Figure M70**  | Uv spectrum of compound **8** | 80 |
| **Figure M71**  | ^1^H NMR spectrum of compound **8** | 81 |
| **Figure M72**  | Expansion of part (δ 5.0 – 1.7) of ^1^H NMR spectrum of compound **8** | 82 |
| **Figure M73**  | Expansion of part (δ 9.1 – 4.8) of ^1^H NMR spectrum of compound **8** | 83 |
| **Figure M74**  | Expansion of part (δ 7.4 – 5.6) of ^1^H NMR spectrum of compound **8** | 84 |
| **Figure M75**  | ESIMS of compound **8** | 85 |
| **Figure M76**  | HR-FAB-MS of compound **8** | 86 |
| **Figure M77**  | IR spectrum of compound **9** | 87 |
| **Figure M78**  | Uv spectrum of compound **9** | 88 |
| **Figure M79**  | ^1^H NMR spectrum of compound **9** | 89 |
| **Figure M80**  | Expansion of part (δ 9.3 – 5.9) of ^1^H NMR spectrum of compound **9** | 90 |

| **Figure M81**  | Expansion of part (δ 5.0 – 1.7) of ^1^H NMR spectrum of compound **9** | 91 |
| --- | --- | --- |
| **Figure M82**  | ESIMS of compound **9** | 92 |
| **Figure M83**  | HR-FAB-MS of compound **9** | 93 |
| **Figure M84**  | IR spectrum of compound **10** | 94 |
| **Figure M85**  | Uv spectrum of compound **10** | 95 |
| **Figure M86**  | ^1^H NMR spectrum of compound **10** | 96 |
| **Figure M87**  | Expansion of part (δ 9.3 – 7.1) of ^1^H NMR spectrum of compound **10** | 97 |
| **Figure M88**  | Expansion of part (δ 6.7 – 4.8) of ^1^H NMR spectrum of compound **10** | 98 |
| **Figure M89**  | Expansion of part (δ 3.7 – 1.7) of ^1^H NMR spectrum of compound **10** | 99 |
| **Figure M90**  | ESIMS of compound **10** | 100 |
| **Figure M91**  | HR-FAB-MS of compound **10** | 101 |
| **Figure M92**  | IR spectrum of compound **11** | 102 |
| **Figure M93**  | Uv spectrum of compound **11** | 103 |
| **Figure M94**  | ^1^H NMR spectrum of compound **11** | 104 |
| **Figure M95**  | Expansion of part (δ 9.5 – 5.9) of ^1^H NMR spectrum of compound **11** | 105 |
| **Figure M96**  | Expansion of part (δ 7.5 – 5.9) of ^1^H NMR spectrum of compound **11** | 106 |
| **Figure M97**  | Expansion of part (δ 5.0 – 1.7) of ^1^H NMR spectrum of compound **11** | 107 |

| **Figure M98**  | ESIMS of compound **11** | 108 |
| --- | --- | --- |
| **Figure M99**  | HR-FAB-MS of compound **11** | 109 |
| **Figure M100**  | IR spectrum of compound **12** | 110 |
| **Figure M101** | Uv spectrum of compound **12** | 111 |
| **Figure M102** | ^1^H NMR spectrum of compound **12** | 112 |
| **Figure M103** | Expansion of part (δ 9.8 – 7.0) of ^1^H NMR spectrum of compound **12** | 113 |
| **Figure M104** | Expansion of part (δ 7.6 – 5.8) of ^1^H NMR spectrum of compound **12** | 114 |
| **Figure M105** | Expansion of part (δ 5.0 – 1.8) of ^1^H NMR spectrum of compound **12** | 115 |
| **Figure M106** | ESIMS of compound **12** | 116 |
| **Figure M107** | HR-FAB-MS of compound **12** | 117 |
| **Figure M108** | IR spectrum of compound **13** | 118 |
| **Figure M109** | Uv spectrum of compound **13** | 119 |
| **Figure M110** | ^1^H NMR spectrum of compound **13** | 120 |
| **Figure M111** | Expansion of part (δ 10.0 – 7.5) of ^1^H NMR spectrum of compound **13** | 121 |
| **Figure M112** | Expansion of part (δ 7.3 – 5.9) of ^1^H NMR spectrum of compound **13** | 122 |
| **Figure M113** | Expansion of part (δ 6.5 – 5.9) of ^1^H NMR spectrum of compound **13** | 123 |
| **Figure M114** | ESIMS of compound **13** | 124 |

| **Figure M115** | HR-FAB-MS of compound **13** | 125 |
| --- | --- | --- |
| **Figure M116** | IR spectrum of compound **14** | 126 |
| **Figure M117** | Uv spectrum of compound **14** | 127 |
| **Figure M118** | ^1^H NMR spectrum of compound **14** | 128 |
| **Figure M119** | Expansion of part (δ 7.3 – 2.8) of ^1^H NMR spectrum of compound **14** | 129 |
| **Figure M120** | Expansion of part (δ 7.3 – 1.7) of ^1^H NMR spectrum of compound **14** | 130 |
| **Figure M121** | FAB-MS of compound **14** | 131 |
| **Figure M122** | HR-FAB-MS of compound **14** | 132 |
| **Figure M123** | IR spectrum of compound **15** | 133 |
| **Figure M124** | Uv spectrum of compound **15** | 134 |
| **Figure M125** | ^1^H NMR spectrum of compound **15** | 135 |
| **Figure M126** | Expansion of part (δ 7.3 – 4.8) of ^1^H NMR spectrum of compound **15** | 136 |
| **Figure M127** | Expansion of part (δ 3.8 – 1.7) of ^1^H NMR spectrum of compound **15** | 137 |
| **Figure M128** | ESIMS of compound **15** | 138 |
| **Figure M129** | HR-FAB-MS of compound **15** | 139 |
| **Figure M130** | IR spectrum of compound **16** | 140 |
| **Figure M131** | Uv spectrum of compound **16** | 141 |

| **Figure M132** | ^1^H NMR spectrum of compound **16** | 142 |
| --- | --- | --- |
| **Figure M133** | Expansion of part (δ 7.7 – 4.8) of ^1^H NMR spectrum of compound **16** | 143 |
| **Figure M134** | Expansion of part (δ 3.7 – 1.7) of ^1^H NMR spectrum of compound **16** | 144 |
| **Figure M135** | ESIMS of compound **16** | 145 |
| **Figure M136** | HR-FAB-MS of compound **16** | 146 |
| **Figure M137** | IR spectrum of compound **17** | 147 |
| **Figure M138** | Uv spectrum of compound **17** | 148 |
| **Figure M139** | ^1^H NMR spectrum of compound **17** | 149 |
| **Figure M140** | Expansion of part (δ 7.7 – 5.9) of ^1^H NMR spectrum of compound **17** | 150 |
| **Figure M141** | Expansion of part (δ 5.0 – 1.7) of ^1^H NMR spectrum of compound **17** | 151 |
| **Figure M142** | ESIMS of compound **17** | 152 |
| **Figure M143** | HR-FAB-MS of compound **17** | 153 |

**Figure M1.** IR spectrum of compound **1**.

11

**Figure M2.** UV spectrum of compound **1**.

12

**Figure M3.** ^1^H NMR spectrum of compound **1**.

13

**Figure M10.** UV spectrum of compound **2**.

**Figure M11.** ^1^H NMR spectrum of compound **2**.

**Figure M14.** ESIMS of compound **2**.

**Figure M15.** HR-FAB-MS of compound **2**.

**Figure M16.** IR spectrum of compound **3**.

**Figure M17.** UV spectrum of compound **3**.

**Figure M18.** ^1^H NMR spectrum of compound **3**.

**Figure M19.** Expansion of part (δ 7.4 – δ 4.8) of ^1^H NMR spectrum of compound **3**.

**Figure M20.** Expansion of part (δ 3.6 – δ 1.7) of ^1^H NMR spectrum of compound **3**.

**Figure M21.** ^13^C NMR spectrum of compound **3**.

40

**Figure M30.** COSY NMR spectrum of compound **3**.

**Figure M34.** HSQC NMR spectrum of compound **3**.

**Figure M45.** NOESY NMR spectrum of compound **3**.

**Figure M46.** Expansion of NOESY NMR spectrum of compound **3**.

**Figure M37.** ESIMS of compound **3**.

**Figure M38.** Expansion of ESIMS of compound **3**.	_48_

**Figure M39.** HR-FABMS of compound **3**.

49

-

**Figure M40.** IR spectrum of compound **4**.	_50_

**Figure M41.** UV spectrum of compound **4**.

51

**Figure M42.** ^1^H NMR spectrum of compound **4**.

**Figure M43.** Expansion of part (δ 7.6 – δ 4.8) of ^1^H NMR spectrum of compound **4**.

**Figure M44.** Expansion of part (δ 3.9 – δ 1.8) of ^1^H NMR spectrum of compound **4**.

**Figure M47.** HR-FAB-MS of compound **4**.

**Figure M49.** UV spectrum of compound **5**.	^59^

**Figure M50.** ^1^H NMR spectrum of compound **5**.

**Figure M51.** Expansion of part (δ 7.5 – δ 4.8) of ^1^H NMR spectrum of compound **5**.

**Figure M52.** Expansion of part (δ 3.6 – δ 1.7) of ^1^H NMR spectrum of compound **5**.

**Figure M58.** ^1^H NMR spectrum of compound **6**.

**Figure M59.** Expansion of part (δ 9.5 – δ 5.9) of ^1^H NMR spectrum of compound **6**.

**Figure M60.** Expansion of part (δ 5.0 – δ 1.8) of ^1^H NMR spectrum of compound **6**.

**Figure M61.** ESIMS of compound **6**.

**Figure M62.** HR-FAB-MS of compound **6**.

**Figure M63.** IR spectrum of compound **7**.

**Figure M64.** UV spectrum of compound **7**.

**Figure M65.** ^1^H NMR spectrum of compound **7**.

**Figure M66.** Expansion of part (δ 7.3 – δ 5.9) of ^1^H NMR spectrum of compound **7**.

**Figure M71.** ^1^H NMR spectrum of compound **8**.

**Figure M72.** Expansion of part (δ 5.0 – δ 1.7) of ^1^H NMR spectrum of compound **8**.

**Figure M73.** Expansion of part (δ 9.1 – δ 4.8) of ^1^H NMR spectrum of compound **8**.

**Figure 74.** Expansion of part (δ 7.4 – δ 5.6) of ^1^H NMR spectrum of compound **8**.

**Figure M79.** ^1^H NMR spectrum of compound **9**.

**Figure M80.** Expansion of part (δ 9.3 – δ 5.9) of ^1^H NMR spectrum of compound **9**.

**Figure M81.** Expansion of part (δ 5.0 – δ 1.7) of ^1^H NMR spectrum of compound **9**.

92

**Figure M82.** ESIMS of compound **9**.

**Figure M83.** HR-FAB-MS of compound **9**.	_93_

**Figure M84.** IR spectrum of compound **10**.	^94^

**Figure M85.** Uv spectrum of compound **10**.

95

**Figure M86.** ^1^H NMR spectrum of compound **10**.

**Figure M87.** Expansion of part (δ 9.3 – δ 7.1) of ^1^H NMR spectrum of compound **10**.

**Figure M88.** Expansion of part (δ 6.7 – δ 4.8) of ^1^H NMR spectrum of compound **10**.

**Figure M89.** Expansion of part (δ 3.7 – δ 1.7) of ^1^H NMR spectrum of compound **10**.

**Figure M90.** ESIMS of compound **10**.

**Figure M91.** HR-FAB-MS of compound **10**.

**Figure M92.** IR spectrum of compound **11**.

**Figure M93.** UV spectrum of compound **11**.

**Figure M94.** ^1^H NMR spectrum of compound **11**.

**Figure M92.** Expansion of part (δ 9.5 – δ 5.9) of ^1^H NMR spectrum of compound **11**.

**Figure M96.** Expansion of part (δ 7.5 – δ 5.9) of ^1^H NMR spectrum of compound **11**.

**Figure M97.** Expansion of part (δ 5.0 – δ 1.7) of ^1^H NMR spectrum of compound **11**.

**Figure M98.** ESI of compound **11**.

**Figure M99.** HR-FAB-MS of compound **11**.

**Figure M100.** IR spectrum of compound **12**.

**Figure M101.** UV spectrum of compound **12**.

**Figure M102.** ^1^H NMR spectrum of compound **12**.

**Figure M103.** Expansion of part (δ 9.8 – δ 7.0) of ^1^H NMR spectrum of compound **12**.

**Figure M104.** Expansion of part (δ 7.6 – δ 5.8) of ^1^H NMR spectrum of compound **12**.

**Figure M105.** Expansion of part (δ 5.0 – δ 1.8) of ^1^H NMR spectrum of compound **12**.

**Figure M106.** ESIMS of compound **12**.

**Figure M107.** HR-FAB-MS of compound **12**.

**Figure M108.** IR spectrum of compound **13**.

**Figure M109.** UV spectrum of compound **13**.

**Figure M110.** ^1^H NMR spectrum of compound **13**.

**Figure M111.** Expansion of part (δ 10.0 – δ 7.5) of ^1^H NMR spectrum of compound **13**.

**Figure M112.** Expansion of part (δ 7.3 – δ 5.9) of ^1^H NMR spectrum of compound **13**.

**Figure M113.** Expansion of part (δ 6.5 – δ 5.9) of ^1^H NMR spectrum of compound **13**.

**Figure M114.** ESIMS of compound **13**.

**Figure M115.** HR-FAB-MS of compound **13**.

**Figure M116.** IR spectrum of compound **14**.

**Figure M117.** Uv spectrum of compound **14**.

**Figure M118.** ^1^H NMR spectrum of compound **14**.

**Figure M119.** Expansion of part (δ 7.3 – δ 2.8) of ^1^H NMR spectrum of compound **14**.

**Figure M120.** Expansion of part (δ 7.3 – δ 1.7) of ^1^H NMR spectrum of compound **14**.

**Figure M121.** FAB-MS of compound **14**.

**Figure M122.** HR-FAB-MS of compound **14**.

**Figure M123.** IR spectrum of compound **15**.

**Figure M124.** Uv spectrum of compound **15**.

**Figure M125.** ^1^H NMR spectrum of compound **15**.

**Figure M126.** Expansion of part (δ 7.3 – δ 4.8) of ^1^H NMR spectrum of compound **15**.

**Figure M127.** Expansion of part (δ 3.8 – δ 1.7) of ^1^H NMR spectrum of compound **15**.

**Figure M128.** ESI of compound **15**.

**Figure M129.** HR-FAB-MS of compound **15**.

**Figure M130.** IR spectrum of compound **16**.

**Figure M131.** UV spectrum of compound **16**.

**Figure M132.** ^1^H NMR spectrum of compound **16**.

**Figure M133.** Expansion of part (δ 7.7 – δ 4.8) of ^1^H NMR spectrum of compound **16**.

**Figure M134.** Expansion of part (δ 3.7 – δ 1.7) of ^1^H NMR spectrum of compound **16**.

**Figure M135.** ESIMS of compound **16**.

**Figure M136.** HR-FAB-MS of compound **16**.

**Figure M137.** IR spectrum of compound **17**.

**Figure M138.** UV spectrum of compound **17**.

**Figure M139.** ^1^H NMR spectrum of compound **17**.

**Figure M140.** Expansion of part (δ 7.7 – δ 5.9) of ^1^H NMR spectrum of compound **17**.

**Figure M141.** Expansion of part (δ 5.0 – δ 1.7) of ^1^H NMR spectrum of compound **17**.

**Figure M142.** ESIMS of compound **17**.

**Figure M143.** HR-FAB-MS of compound **17**.
