# Supplementary material for: Carbothioamide functionalized paroxetine analogues as antiparasitic agents targeting the hypoxanthine-guanine-xanthine phosphoribosyltransferase enzyme: Synthesis, characterization, biochemical, biophysical, and in silico studies
Source: PLoS One. 2026 Oct 22;21(10):e0359519. doi: 10.1371/journal.pone.0359519 (PMC13690214; doi:10.1371/journal.pone.0359519)
Supplement: Supplementary file 3 [file pone.0359519.s002.docx]

**Fig. S1: STD-NMR Analysis of compound 4**. a) ^1^H-NMR (off-resonance) spectrum (blue) of compound 1.b) STD difference spectrum in the presence of HGXPRT protein (red).

**Fig. S2: STD-NMR Analysis of compound 6**. a) ^1^H-NMR (off-resonance) spectrum (blue) of compound 1.b) STD difference spectrum in the presence of HGXPRT protein (red).

**Figure-S3:** 3D Docked poses of compounds (a) **5**, (b) **6**, (c) **7**, and (d) **8** in the allosteric site of HGXPRT. Black color represents hydrogen bond, blue color indicates aromatic hydrogen bond, while cyan color indicates π-π stacking interactions, while green color indicates π-cationic interactions.

**Figure-S4:** 3D Docked poses of compounds (a) **9**, (b) **10**, (c) **11**, and (d) **12** in the allosteric site of HGXPRT. Black color represents hydrogen bond, blue color indicates aromatic hydrogen bond, while cyan color indicates π-π stacking interactions, while green color indicates π-cationic interactions and magenta color indicates salt bridges.

**Figure-** **S5:** 3D Docked poses of compounds (a) **13**, (b) **14**, (c) **15**, (d) **16** and (e) **17** in the allosteric site of HGXPRT. Black color represents hydrogen bond, blue color indicates aromatic hydrogen bond, while cyan color indicates π-π stacking interactions, while green color indicates π-cationic interactions and magenta color indicates salt bridges.

**.**

**Figure-S6:** Ligand properties of (A) standard inhibitor of 3OZF (B) Compound **2** (C) Compound **10** (D) Compound **12** (i) Ligand RMSD (ii) Radius of gyration (rGyr) (iii) Intramolecular hydrogen bonds (intraHB) (iv) Molecular surface area (MolSA) (v) Solvent accessible surface area (SASA) (vi) Polar surface area (PSA).

**Figure-S7**: (A) RMSD graph illustrating the interaction between a protein and standard 9-Deazaguanine. (B) Over the course of a simulation lasting up to 160 ns, residues in the interaction histogram display a variety of non-covalent interactions, indicating a constant binding affinity.

**Interaction fraction**

**Bars of residues in interaction**

**Figure-S8**: (A) protein ligand RMSD graph of compound **2** (MIA 20). (B) residues in interaction histogram showing various non-covalent interactions for upto 80ns simulation time indicative of stable interactions within binding pocket.

**Interaction fraction**

**Bars of residues in interaction**

**Figure-S9:** (A) RMSD graph showing how the compound **10** interacts with a protein in a site 3. (B) Residues in the interaction histogram exhibit a range of non-covalent interactions during a simulation duration of up to 160 ns, suggesting a consistent binding affinity throughout the simulation.

**Interaction fraction**

**Bars of residues in interaction**

**Figure-S10:** (A) RMSD graph for the protein-ligand of the compound **12** (mia15). (B) Residues in the interaction histogram illustrate different non-covalent interactions for up to 160 ns simulation duration, suggesting stable binding affinity throughout the simulation

**Interaction fraction**

**Bars of residues in interaction**

**Table S1:** Synthetic details of Compounds (**1**-**17**)

| **Compound** | **Physical State/ Appearance** | **Percent Yield** | **Retention Factor** |
| --- | --- | --- | --- |
| **1** | Dull white solid | 91 % | R_f_ = 0.75 |
| **2** | White solid | 46% | R_f_ = 0.42 |
| **3** | White solid | 61% | R_f_ = 0.48 |
| **4** | White solid | 62 % | R_f_ =0.42 |
| **5** | Yellow solid | 96 % | R_f_ =0.61 |
| **6** | White solid | 69% | R_f_ = 0.42 |
| **7** | White solid | 72 % | R_f_ = 0.6 |
| **8** | White solid | 70 % | R_f_ = 0.6 |
| **9** | White solid | 65% | R_f_ = 0.35 |
| **10** | Light yellow solid | 96 % | R_f_ =0.42 |
| **11** | Light yellow solid | 74 % | R_f_ = 0.33 |
| **12** | Yellow solid | 68% | R_f_ = 0.26 |
| **13** | Yellow solid | 89% | R_f_ = 0.54 |
| **14** | Dull white solid | 72 % | R_f_ =0.48 |
| **15** | White solid | 50% | R_f_ =0.35 |
| **16** | Light yellow solid | 75 % | R_f_ =0.42 |
| **17** | Light yellow solid | 91% | R_f_ = 0.51 |

| Table S2: SiteMap analysis of 3OZF protein. | | | | |
| --- | --- | --- | --- | --- |
| Binding Sites | **SiteScore** | **Size** | **Dscore** | **Volume** |
| Site 1 | 0.907 | 1189 | 1.017 | 795.074 |
| Site 2 | 0.903 | 1131 | 1.011 | 802.620 |
| Site 3 | 0.901 | 906 | 0.974 | 724.073 |
| Site 4 | 0.871 | 286 | 0.989 | 183.505 |
| Site 5 | 0.866 | 273 | 0.977 | 181.104 |

**Table S3**: MMGBSA Analysis of compounds **1-17**

| **Compound** | **Docking score** | **MMGBSA ΔG Bind** | **MMGBSA ΔG Bind Coulomb** | **MMGBSA ΔG Bind Covalent** | **MMGBSA ΔG Bind Hbond** | **MMGBSA ΔG Bind Lipo** | **MMGBSA ΔG Bind vdW** |
| --- | --- | --- | --- | --- | --- | --- | --- |
| 1 | -2.359 | -25.05 | -9.22 | 3.76 | 0 | -16.39 | -43.14 |
| 2 | -1.87 | -13.3 | -5.9 | 10.23 | -0.19 | -10.48 | -41.19 |
| 3 | -1.293 | -17.69 | -8.45 | 10.13 | -0.25 | -10.33 | -47.75 |
| 4 | -2.387 | -5.88 | 1.99 | 3.45 | -0.46 | -11.74 | -38.76 |
| 5 | -1.57 | -6.97 | -12.29 | 16.01 | -0.24 | -12.01 | -43.84 |
| 6 | -0.233 | -26.27 | -5.3 | 5.02 | -0.34 | -15.12 | -43.38 |
| 7 | -1.136 | -5.84 | -6.87 | 0.82 | 0 | -11.05 | -37.2 |
| 8 | -2.781 | -16.37 | -14.78 | 5.36 | -0.07 | -11.64 | -38.67 |
| 9 | -2.266 | -15.05 | -2.31 | 6.19 | 0 | -13.52 | -41.8 |
| 10 | -1.637 | -9.16 | -17.86 | 8.64 | -1.4 | -7.54 | -33.79 |
| 11 | -0.609 | -13.73 | -5.19 | 10.38 | -0.99 | -15.29 | -38.76 |
| 12 | -2.56 | -17.54 | -5.54 | 1.86 | -0.01 | -11.8 | -32.71 |
| 13 | -0.928 | -17.19 | -4.03 | 0.59 | -0.57 | -8.99 | -32.7 |
| 14 | -1.086 | -18.44 | -9.73 | -2.7 | -0.37 | -7.04 | -30.89 |
| 15 | -1.527 | -25.3 | -13.77 | 8.54 | -0.2 | -13.89 | -44.45 |
| 16 | -1.633 | -7.2 | -7.64 | -3.52 | -0.66 | -4.51 | -26.2 |
| 17 | -1.949 | -27.39 | -12.16 | -1.89 | -0.06 | -13.4 | -37.13 |
| Standard Inhibitor (9- Deazaguanine) | -8.348 | 36.29 | -37.61 | 2.12 | -4.67 | -1.73 | -23.72 |

|  |  |  | **Table S4: ADMET Profile using Maestro QikProp tool** | | | | |  |  |  |  |  |  |  |
| --- | --- | --- | --- | --- | --- | --- | --- | --- | --- | --- | --- | --- | --- | --- |
|  |  |  |  |  |  |  |  |  |  |  |  |  |  |  |
|  | A | B | C | D | E | F | G | H | I | J | K |  |  |  |
| **Entry ID** | **mol MW** | **donorHB** | **accptHB** | **LIPINSKI'S RULE VIOLATIONS (RULEOFFIVE)** | **OCTANOL/WATER – LIPOPHILICITY QPLOGPO/W** | **PREDICTED HERG K+ CHANNEL BLOCKAGE QPLOGHERG** | **CACO-2 CELL PERMEABILITY (NM/SEC) QPPCACO** | **BRAIN/BLOOD PARTITION COEFFICIENT QPLOGBB** | **MDCK CELL PERMEABILITY (NM/SEC) QPPMDCK** | **SKIN PERMEABILITY (LOG KP, CM/S) (QPLOGKP)** | **Plasma Protein Binding (QPlogKhsa)** | **Interpretation of 11 parameters (Score out of 11)** | **% ADMET PARAMETERS** | **safe profile %** |
| **Reference range** | **130–725 Da** | **0–6** | **2–20** | **0–1 violation (≤1 preferred)** | **–2.0 to 6.5** | **> –5.0 (less negative is better for safety)** | **< 25 poor, > 500 great** | **–3.0 to 1.2** | **< 25 poor, > 500 great** | **–8.0 to –1.0** | **-1.5 to 1.5** |  |  |  |
| 1 | 464.553 | 1 | 4.75 | 1 | 6.844 | -6.666 | 9509.51 | 0.552 | 10000 | 0.388 | 1.27 | 2 | 18.18181818 | 81.81818182 |
| 2 | 498.999 | 1 | 4.75 | 1 | 6.773 | -5.545 | 8190.547 | 0.627 | 10000 | -0.062 | 1.224 | 1 | 9.090909091 | 90.90909091 |
| 3 | 498.999 | 1 | 4.75 | 1 | 7.311 | -7.057 | 8119.281 | 0.62 | 10000 | 0.183 | 1.399 | 2 | 18.18181818 | 81.81818182 |
| 4 | 533.444 | 1 | 4.75 | 2 | 7.682 | -6.383 | 8406.999 | 0.805 | 10000 | -0.048 | 1.487 | 2 | 18.18181818 | 81.81818182 |
| 5 | 533.444 | 1 | 4.75 | 2 | 7.192 | -5.941 | 7942.425 | 0.729 | 10000 | -0.143 | 1.33 | 2 | 18.18181818 | 81.81818182 |
| 6 | 543.45 | 1 | 4.75 | 2 | 7.072 | -6.223 | 9224.382 | 0.721 | 10000 | 0.103 | 1.289 | 3 | 27.27272727 | 72.72727273 |
| 7 | 482.544 | 1 | 4.75 | 1 | 6.343 | -5.072 | 9478.952 | 0.678 | 10000 | -0.085 | 1.05 | 0 | 0 | 100 |
| 8 | 482.544 | 1 | 4.75 | 1 | 6.916 | -6.511 | 8293.075 | 0.543 | 10000 | 0.188 | 1.297 | 2 | 18.18181818 | 81.81818182 |
| 9 | 482.544 | 1 | 4.75 | 1 | 6.742 | -6.177 | 9392.052 | 0.623 | 10000 | 0.193 | 1.206 | 2 | 18.18181818 | 81.81818182 |
| 10 | 500.535 | 1 | 4.75 | 2 | 6.846 | -6.031 | 9660.025 | 0.708 | 10000 | 0.144 | 1.21 | 3 | 27.27272727 | 72.72727273 |
| 11 | 500.535 | 1 | 4.75 | 2 | 6.358 | -4.636 | 7125.916 | 0.681 | 10000 | -0.476 | 1.043 | 2 | 18.18181818 | 81.81818182 |
| 12 | 509.551 | 1 | 5.75 | 2 | 5.772 | -6.147 | 1026.782 | -0.531 | 1750.017 | -1.717 | 1.098 | 1 | 9.090909091 | 90.90909091 |
| 13 | 509.551 | 1 | 5.75 | 2 | 5.215 | -5.049 | 781.31 | -0.539 | 1266.785 | -2.198 | 0.946 | 1 | 9.090909091 | 90.90909091 |
| 14 | 548.551 | 1 | 4.75 | 2 | 7.859 | -6.837 | 9189.881 | 0.737 | 10000 | 0.272 | 1.517 | 3 | 27.27272727 | 72.72727273 |
| 15 | 494.58 | 1 | 5.5 | 1 | 6.501 | -5.787 | 9245.081 | 0.483 | 10000 | 0.109 | 1.12 | 2 | 18.18181818 | 81.81818182 |
| 16 | 532.552 | 1 | 4.75 | 2 | 7.639 | -6.34 | 9446.775 | 0.812 | 10000 | 0.106 | 1.478 | 3 | 27.27272727 | 72.72727273 |
| 17 | 489.563 | 1 | 6.25 | 1 | 6.022 | -6.568 | 1849.514 | -0.283 | 3380.644 | -1.063 | 1.084 | 0 | 0 | 100 |
| 9-Deazaguanine.1 | 150.14 | 4 | 4 | 0 | -0.295 | -3.304 | 128.028 | -1.042 | 53.635 | -4.658 | -0.746 | 2 | 18.18181818 | 81.81818182 |

Table S5: Percent cell viability in the presence of active compounds

| **Compound Number** | **Percent cell viability ±SEM^a^ (μM)** |
| --- | --- |
| Untreated Cells | 99 ± 4.9 |
| **1** | 95.43 ± 2.1 |
| **2** | 91.5 ± 3.78 |
| **3** | 97.1 ± 0.7 |
| **4** | 86.60 ± 8.4 |
| **5** | 83.90 ± 7.9 |
| **6** | 71.90 ± 2.69 |
| **7** | 94.6 ± 1.9 |
| **8** | 98.7 ± 2.8 |
| **9** | 83.39 ± 3.18 |
| **10** | 81.4 ± 5.1 |
| **11** | 95.4 ± 0.01 |
| **12** | 95.1 ± 0.1 |
| **13** | 95.3 ± 0.01 |
| **14** | 95.51 ± 0.09 |
| **15** | 95.690 ± 0.02 |
| **16** | 95.47 ± 0.02 |
| **17** | 95.10 ± 0.08 |

SEM^a^ is the Standard Error of the Mean.

**Control STD NMR Experiment in the absence of protein**

In the control experiment the compounds were run alone for the STD-NMR experiment to rule out the false negative STD effects. As per suggestion of the reviewer we have presented the spectra for compound **2**.

S Figure A: STD NMR spectra for the compound **2** (a) off-resonance 1D NMR spectrum (I*_off_*) in the absence of enzyme. (b) On- resonance 1D NMR spectrum (I*_on_*) in the absence of enzyme. (c) STD difference spectra of compound **2**.  Ligand (1 mM), on-, and off-resonance frequencies were -134 and 18,000 Hz, respectively, Relaxation delay (d1) was 3.1 s, saturation time (d20) was 3 seconds, and number of scans were 1024.

**STD NMR Experiment in the Presence of Protein**

In the experiment the compounds were run in the presence of protein for the STD-NMR experiment to see STD effects originated because of protein. As per suggestion of the reviewer we have presented the spectra for compound **2**.

S Figure B: STD NMR spectra for the compound **2** (a) off-resonance 1D NMR spectrum (I*_off_*) in the presence of enzyme. (b) On- resonance 1D NMR spectrum (I*_on_*) in the presence of enzyme. (c) STD difference spectra of compound **2**.  Ligand to protein ratio was 100:1, on-, and off-resonance frequencies were -134 and 18,000 Hz, respectively, Relaxation delay (d1) was 3.1 s, saturation time (d20) was 3 seconds, and number of scans were 1024.

Raw Data of the STD NMR for the compound **2**

a

b

S Figure C: a) Enzyme activity in the presence of buffer, b) Enzyme activity in the presence of 1 (blue), 5 (green), and 10 % (red) of DMSO.

**S Figure D: Dose response curve for the standard compound (a) and identified inhibitors (b-r).**

Table S6: Structured comparison of known HGXPRT inhibitor

| **Inhibitor class** | **Representative examples** | **Main limitations (including toxicity risks)** |
| --- | --- | --- |
| Purine base analogs | 6-mercaptopurine, 6-thioxanthine, allopurinol-related purines | Limited selectivity; host purine metabolism effects; variable potency; rapid metabolism |
| 9-Deazapurines / modified purines | 9-deazaxanthine, substituted guanine analogs | Solubility issues; species-dependent activity; synthesis complexity |
| Acyclic nucleoside phosphonates | Purine phosphonate analogs | Poor membrane permeability due to negative charge; prodrug often needed; renal/toxicity concerns possible |
| Transition-state analogs | Immucillin-like / DADMe-style analogs | Complex synthesis; oral PK challenges; possible off-target salvage enzyme binding |
| PRPP-site inhibitors | Ribose-phosphate / pyrophosphate mimics | Highly polar; weak cellular uptake; nonselective against related phosphoribosyltransferases |
| Bisubstrate analogs | Purine linked to phosphate/ribose motifs | Large size, low permeability, synthetic difficulty |
| **Inhibitors identified in the current study** | | |
| Paroxetine-carbothioamide derivatives | The compounds are structurally distinct from classical purine analogs as incorporation of a carbothioamide linkage and substituted phenyl ring expands chemical space. Unlike purine analogs (which act via substrate mimicry), these compounds likely to interact through alternative binding modes (supported by docking). This includes interaction with hydrophobic and peripheral active-site regions. The scaffold used is based on a drug-like framework (paroxetine-derived) therefore it will reduce polarity compared to purine analogs, it will potentially increase membrane permeability. | |
